# A High-Throughput Method for Illumina RNA-Seq Library Preparation

**DOI:** 10.3389/fpls.2012.00202

**Published:** 2012-08-28

**Authors:** Ravi Kumar, Yasunori Ichihashi, Seisuke Kimura, Daniel H. Chitwood, Lauren R. Headland, Jie Peng, Julin N. Maloof, Neelima R. Sinha

**Affiliations:** ^1^Department of Plant Biology, University of CaliforniaDavis, CA, USA; ^2^Department of Statistics, University of CaliforniaDavis, CA, USA

**Keywords:** cDNA fragmentation, high-throughput, Illumina, mRNA isolation, multiplexing, RNA-Seq, sequencing

## Abstract

With the introduction of cost effective, rapid, and superior quality next generation sequencing techniques, gene expression analysis has become viable for labs conducting small projects as well as large-scale gene expression analysis experiments. However, the available protocols for construction of RNA-sequencing (RNA-Seq) libraries are expensive and/or difficult to scale for high-throughput applications. Also, most protocols require isolated total RNA as a starting point. We provide a cost-effective RNA-Seq library synthesis protocol that is fast, starts with tissue, and is high-throughput from tissue to synthesized library. We have also designed and report a set of 96 unique barcodes for library adapters that are amenable to high-throughput sequencing by a large combination of multiplexing strategies. Our developed protocol has more power to detect differentially expressed genes when compared to the standard Illumina protocol, probably owing to less technical variation amongst replicates. We also address the problem of gene-length biases affecting differential gene expression calls and demonstrate that such biases can be efficiently minimized during mRNA isolation for library preparation.

## Introduction

Recent advances in RNA-sequencing (RNA-Seq) have provided a means for rapid characterization and quantification of transcriptomes. RNA-Seq involves direct sequencing of complementary DNAs (cDNAs) using high-throughput next generation sequencing (NGS) technologies, followed by mapping of the sequencing reads to the reference genome or gene sets for gene expression analysis and polymorphism detection. Compared to other technologies such as hybridization-based microarrays and Sanger sequencing-based methods, RNA-Seq provides a more comprehensive understanding of transcriptome complexity and the ability to detect a dynamic range of expression levels (Marioni et al., 2008; Wang et al., 2009; Mader et al., 2011), allowing for the identification of novel transcripts, small RNAs, SNPs, alternate splicing products, sense and antisense transcripts, fusion transcripts, and can identify transcription initiation sites (Ozsolak and Milos, 2011).

Next generation sequencing platforms used for RNA-Seq are commercially available from Illumina, Roche, ABI, Helicos BioSciences, and more, and companies are continuously improving their platforms to increase sequencing speeds, accuracy, and depth at a lower cost. Cost reduction and high sequencing performance allow for projects such as the 10 million dollar 100 human genomes^1^ and the *Arabidopsis* 1001 genomes project (Weigel and Mott, 2009). Even though sequencing capacity continues to increase, protocols for sample library preparation, being laborious, time consuming, and expensive, remain a limiting step. Sequencing library preparation involves the production of a random collection of sequence-ready adapter-modified DNA fragments, with a specific range of fragment sizes. Although several procedures to improve on the Illumina RNA-Seq library preparation have been published (Quail et al., 2008; Nagalakshmi et al., 2010; Wilhelm et al., 2010), these protocols still have several laborious steps including ethanol precipitation, column purifications, and gel extraction for size fractionation. In addition to being time consuming, these steps carry a high risk of cross-contamination and sample mix-up inherent in protocols involving extensive individual sample handling. Recently, Illumina introduced a high-throughput method (TruSeq RNA sample preparation kit) replacing these purification steps with solid-phase reversible immobilization (SPRI) magnetic bead reaction cleanup methodology (Hawkins et al., 1994; Lennon et al., 2010). Using this method, a single technician can make 96 libraries from total RNA in 3 days. However, the amount of multiplexing is limited to 24 by the number of available barcodes. Similar improvements can also be seen in the protocols by Zhong et al. (2011) and Wang et al. (2011a).

Here we present several improvements to the Illumina sample preparation for RNA-Seq protocol (Illumina Inc., San Diego, USA, Cat. # RS-100-0801) that we have made to generate high-throughput and cost-effective RNA-Seq libraries in a more robust and reproducible way, compared with other current protocols. We integrated a direct mRNA extraction method using Dynabeads oligo dT beads (Invitrogen, Carlsbad, CA, USA) or Sera-Mag oligo dT beads (Thermo Scientific, Indianapolis, IN, USA), which are suitable for RNA extraction from various plant and animal tissues. One challenge for scaling-up protocols to 96-well format is the RNA fragmentation step. Specifically, it is difficult to control the degree of chemical fragmentation in RNA owing to the short incubation time, leading to decreased reproducibility, especially in 96-well formats. To overcome this problem we used enzymatic fragmentation of cDNA. We also used the SPRI magnetic bead reaction cleanup methodology to enable handling of samples in a 96-well format, similar to the TruSeq protocol and that of Zhong et al. (2011). Further, to reduce protocol time and the number of handling steps, we applied an “on beads” protocol (Fisher et al., 2011) for several enzymatic reactions including end repair, A-tailing, and adaptor ligation. These changes reduce the potential for human error introduced during the sample preparation process. Finally, we developed 96 unique barcoded adapters to provide more flexibility in multiplexing. With these modifications and a few other small adjustments, we have greatly increased the efficiency and reproducibility, and lowered the cost of library preparation (by ~3–11×) in comparison to other currently available methods. Our high-throughput RNA-seq (HTR) library preparation method enables a single researcher to reproducibly make 96 RNA-Seq libraries, starting from tissue, in less than 3 days. Analysis of the sequencing output from our libraries demonstrated that our protocol yields data whose quality matches or exceeds that of the standard Illumina method (IL) by sequence composition, ribosomal RNA contamination, and detection of gene expression.

## Materials and Methods

**Figure 1:**
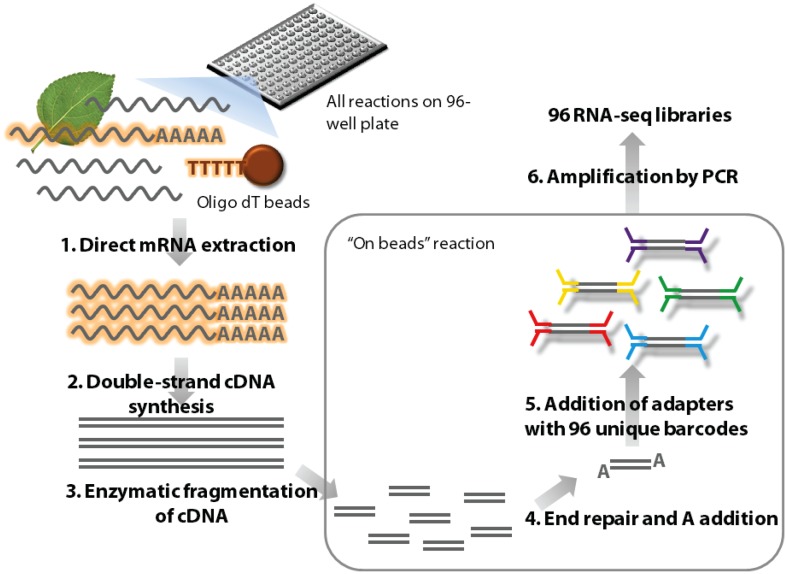
**Outline of the high-throughput RNA-seq (HTR) library preparation**. In short, frozen tissue samples are ground in the lysis buffer and mRNA is isolated from this using oligo dT beads (1). The mRNA is used to make first and second strands of cDNA (2) and this double stranded cDNA molecules are subsequently enzymatically fragmented (3). The ends of these molecules are repaired and an A nucleotide is added (4) to facilitate TA ligation of the barcoded adapters (5). The ligated samples are then enriched by amplification using adapter specific primers (6) and purified for sequencing.

Please see Methods 1 in Supplementary Material for a detailed library synthesis protocol. An outline of our new high-throughput library preparation method (HTR) is given in Figure 1 and a comparative overview with the standard Illumina protocol (IL) is shown in Figure S6 in Supplementary Material.

### Plant materials

Seeds of two tomato species: *Solanum lycopersicum* var. M82 (LA3475) and *S. pennellii* (LA0716) were obtained from the TGRC^2^. For synchronized germination, seeds were treated with 50% household bleach (~2.7% sodium hypochlorite) for 30–60 s, rinsed with water, sown on wet paper towels in petri dishes, and placed in darkness for 3 days. Petri dishes were then exposed to light and grown at 22°C with a day-length of 16 h in a Conviron controlled environment chamber under cool-white lights (95 μE) in a randomized design. Four days after transferring to the light, plants were transplanted to soil. Ten days from transplanting, dissected shoot apices (first leaf and cotyledons removed) were collected with five plants pooled per replicate.

### Summary of library preparation and sequencing

The total tissue collected for each *S. lycopersicum* sample weighed less than 100 mg and each *S. pennellii* sample weighed less than 50 mg (owing to its smaller size). For the Illumina (IL) library preparations, total RNA was first extracted from collected tissues using the Plant RNeasy mini kit (Qiagen) and libraries prepared using mRNA-Seq 8 sample prep kit (Illumina Inc. San Diego, USA, Cat. # RS-100-0801) according to the manufacturer’s protocol. For HTR library preparations, mRNA isolation with both Dynabeads (Invitrogen) and Sera-Mag oligo dT magnetic beads (Thermo Scientific, Cat. # 3815-2103-010150) were performed based on the Dynabeads mRNA direct kit (Invitrogen) protocol with minor adjustments (see Methods 1 in Supplementary Material). The control libraries C1 and C2 were made with total RNA extracted using the Plant RNeasy mini kit (Qiagen) and the mRNA was isolated using a custom protocol (see Methods 2 and 3 in Supplementary Material). All samples were purified and multiplexed using eight of the three-nucleotide barcoded adapters, randomly assigned to three different pools with eight samples per pool (Table S1 in Supplementary Material). These pooled libraries were sequenced at the UC Davis Genome Centre Expression Analysis Core using either Illumina’s GAII sequencing system or the HiSeq 2000 (Illumina Inc. San Diego, CA, USA).

### Tissue disruption in high-throughput RNA-seq

Isolation of RNA from plants usually involves grinding tissue using a mortar and pestle to facilitate cell wall disruption: a time-consuming process that is prone to contamination. To resolve these issues, we used the Mini-beadbeater-96 high-throughput cell disruptor (BioSpec), in which it is possible to process 24 samples at a time in 2 ml tubes. To quickly resuspend tissue and inactivate endogenous RNAses, we added extraction/homogenization buffer to the samples prior to homogenization. Antifoam A was used to prevent foaming that otherwise would impair the proper disruption of tissue. Thus, our protocol allows rapid isolation of mRNA directly from tissue and minimizes the risk of cross-contamination. One major limitation of high-throughput experiments such as RNA-seq is obtaining enough tissue for multiple replicates from certain tissues. However, using our protocol we have successfully produced libraries from less than 5 mg of tissue (Ichihashi Y., Sinha, N. unpublished results), owing to direct mRNA isolation as well as decreased sample handling and transfer steps that typically result in loss of RNA and cDNA.

### mRNA isolation in HTR

We have optimized the mRNA isolation protocol to obtain high quality mRNA that has very little DNA and ribosomal RNA contamination. To accomplish this, we used oligo dT beads for direct mRNA extraction from tissue, rather than extracting total RNA first as done in other established protocols. By extracting mRNA directly from tissue, we decreased sample handling and reduced the number of steps required by 30% in comparison to the IL protocol where total RNA is extracted first.

### cDNA fragmentation in HTR

The Illumina RNA-Seq sample preparation method achieves RNA fragmentation by the use of divalent cations. Although this works well for 6–12 samples, this process is rapid (5–10 min) and prone to over-fragmentation if not well controlled. Therefore it is difficult to use in a high-throughput platform. To overcome these problems, we employed the NEBNext^®^ DNA fragmentase enzyme mix (NEB, Beverley, MA, USA) to cleave double stranded cDNA molecules. The enzymatic process has a somewhat longer incubation time; thus, stopping the reaction is less time-sensitive. This allowed us to obtain more uniform libraries when processing numerous samples. We optimized the conditions such that on average, 300 bp fragments were obtained after digestion (see Methods 4 in Supplementary Material). We determined that digestion of cDNA for 30 min with the NEBNext^®^ DNA fragmentase enzyme mix was an effective alternative to chemical fragmentation of RNA in the range of expected cDNA output from our protocol (100–500 ng) by test digests of 100 and 500 ng of DNA ladder (Figure S2 in Supplementary Material).

### Purification and size exclusion in HTR

Size fractionation by agarose gel electrophoresis and the subsequent gel extraction are among the most time-consuming steps in the Illumina method; furthermore, this method requires purification columns for gel extraction, a procedure not amenable to the 96-well format convenient for high-throughput applications. Therefore, we replaced this step with the use of Ampure XP solid-phase reverse immobilization (SPRI) magnetic beads (Agencourt Bioscience, Beverley, MA, USA), which enabled us to perform all purifications in a 96-well format. By adjusting the amount of polyethylene glycol (PEG) in the incubation buffer, we were able to selectively enrich for library fragments greater than 300 bp, thereby drastically reducing adapter and primer-dimer contamination and eliminating the need for time-consuming gel extraction steps (see Methods 4 in Supplementary Material). SPRI bead based size selection has the added benefit of minimizing the risk of sample mix-up and contamination since the samples stay in the plate in a 96-well format. As shown in Figure S3 in Supplementary Material, the use of PEG-precipitation and magnetic bead purification was an effective alternative to size fractionation by agarose gel electrophoresis and gel extraction. By testing the effects of different concentrations of PEG on size-specific DNA precipitation and purification, we were able to perform some of the library synthesis steps without removing Ampure XP beads prior to the subsequent enzymatic reaction, an “on beads” protocol as described in Fisher et al. (2011). There is a twofold advantage to this “on beads” protocol: cost effectiveness as the beads are being reused, and also reduction in the handling steps, consumables, and potential human error.

### Barcoded adapter design

Previous multiplexing strategies (such as Illumina’s TruSeq sample prep kit and Fox-Walsh et al., 2011) are limited to 24 samples and require an extra index read run because this method adds indices to one of the adapters at the PCR enrichment stage (Meyer and Kircher, 2010). Therefore, similar to some other methods (Craig et al., 2008), we have designed barcoded adapters which are directly read while sequencing and do not require an extra sequencing step. Using python scripts described in a previous published article^3^ (Meyer and Kircher, 2010), we generated 96 unique five nucleotide barcodes and 8 unique three-nucleotide barcodes whereby up to two substitutions in sequencing or PCR errors can be tolerated without mutating the sequence into another barcode (Table S2 in Supplementary Material). The barcodes were selected and ordered in a way that there is an even distribution of nucleotides (20–30% of each nucleotide) in the first three positions when the adapters are sequentially selected in multiples of 16. The barcodes were included in the oligonucleotide sequences (termed PE1 and PE2, Table S2 in Supplementary Material) and were synthesized commercially (Sigma–Aldrich) with PE1 having an added 5′ phosphate. The adapters were then prepared by annealing PE1 and PE2 oligonucleotide pairs using the protocol listed in Methods 4 in Supplementary Material. The barcoded samples exhibit similar percent mapping to reference genes, indicating that they have comparable performance (Tables S1 and S3 in Supplementary Material).

### Bioinformatics

All the bioinformatics and statistical analyses were performed either on our local servers or the iPLANT Atmosphere cloud server (Goff et al., 2011). The 40 bp single end sequence reads obtained were quality trimmed and parsed to individual libraries using custom Perl scripts. Sequence quality estimations, GC content, nucleotide distribution, and read duplication levels were determined for the samples using FASTQC^4^ and the results were plotted using ggplot2 (Wickham, 2009). The reads were mapped to 34,727 tomato cDNA sequences predicted from the gene models from the ITAG2.4 genome build (The Tomato Genome Consortium, 2012; downloadable from http://solgenomics.net/itag/release/2.3/list_files) using bowtie (Langmead et al., 2009) with the following parameters: -e 160 –solexa1.3-quals -a –best –strata -m 1 -n 2 -p 8 –sam –tryhard. The uniquely mapped read data output was processed using custom scripts in Perl and R, then normalized using the Bioconductor package EdgeR ver. 2.2.5 (Robinson and Oshlack, 2010) using the trimmed mean of *M*-values method (Robinson and Oshlack, 2010), whereby scale factors between samples are estimated and used for the statistical analysis. Reads were filtered such that there were at least a sum total of 20 reads across all 16 samples for each gene. Differential expression calls were also made using the EdgeR package. Genes whose adjusted *p*-values (BH method; Benjamini and Hochberg, 1995) was less than 0.01 were considered differentially expressed. All graphs were made using the core R functions (R Development Core Team, 2011), EdgeR and the packages “ggplot2” (Wickham, 2009), and “VennDiagram” (Chen and Boutros, 2011).

## Results and Discussion

To compare our new HTR library preparation method with the standard Illumina (IL) protocol, for each protocol, we evaluated RNA-Seq library reads generated from all four biological replicates of *S. lycopersicum* and *S. pennellii*.

### Read quality

**Figure 2:**
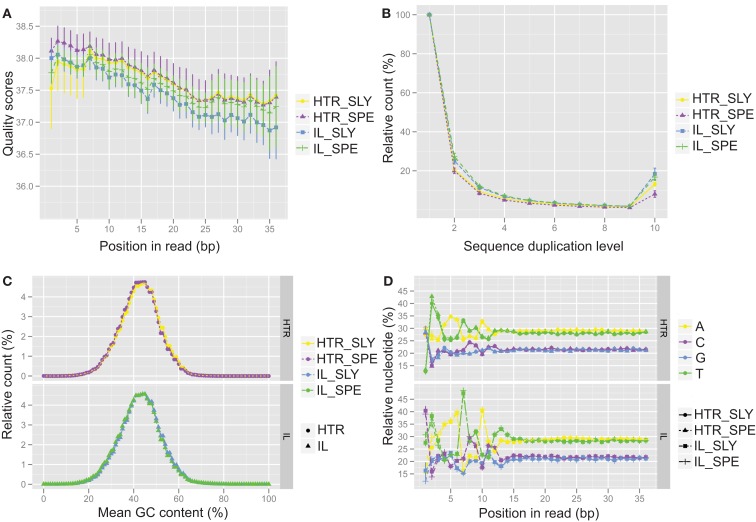
**Quality control analysis for Illumina (IL) and high-throughput RNA-seq (HTR) library preparations**. The quality control data from IL and HTR protocols using *S. lycopersicum* (SLY) and *S. pennellii* (SPE) are shown. **(A)** Per base sequence quality. Average of the four replicates has been plotted here. Error bars represent SD. **(B)** Sequence duplication levels. **(C)** Per sequence GC content. **(D)** Per base sequence content. In **(C)** and **(D)**, the SPE and SLY of HTR protocol are plotted in the top panel and SPE and SLY of IL protocol are plotted in the bottom panel. Graphs were made in R using ggplot2.

**Figure 3:**
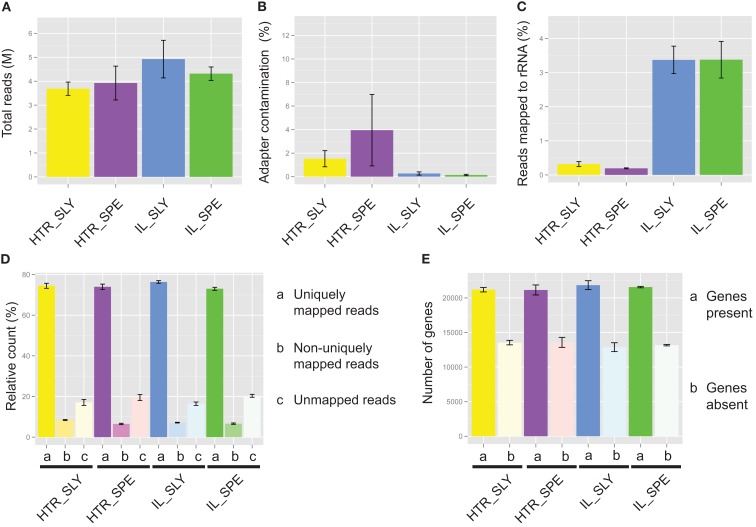
**Read mapping for Illumina (IL) and high-throughput RNA-seq (HTR) library preparations**. **(A)** Total number of reads. **(B)** Adapter contamination. **(C)** rRNA contamination. **(D)** Percentage reads mapped. **(E)** Number of detected genes. The read mapping data from IL and HTR protocols using *S. lycopersicum* (SLY) and *S. pennellii* (SPE) are shown. Graphs were made in R using ggplot2. Error bars are ±SEM.

A comparison of several statistical parameters on the data generated from HTR and IL protocol libraries showed that our HTR method produced RNA-Seq data is of similar quality to that of the IL protocol. We compared reads obtained from both protocols, and found that the qualities of reads along nucleotide position were equivalent (Figure 2A) and that greater than 97% of the reads passed the quality filters (Table S4 in Supplementary Material). The number of duplicated reads was also similar for both methods (Figure 2B), showing that our HTR protocol does not produce an overabundance of amplification artifacts. Similar GC content was observed in both methods indicating that HTR does not show a greater GC bias when compared to IL (Figure 2C). Unsurprisingly, nucleotide distribution bias was observed in the first 8–12 bases of samples prepared using either technique (Figure 2D). This is caused by the use of random primers to generate cDNA for RNA-Seq experiments. There are biases in these “random” primer populations that can lead to reverse transcription of certain regions at higher levels than others (Hansen et al., 2010). The total number of reads obtained was also similar for both methods (Figure 3A), indicating that libraries from our HTR protocol were well-incorporated into the Illumina flow cell for cluster generation and sequencing.

### Adapter contamination

Most of the samples had low levels of adapter contamination, indicating that the size exclusion by SPRI beads works well (Figure 3B).

### Percentage rRNA contamination

On average IL showed 5% and HTR method showed less than 0.5% rRNA contamination (Figure 3C), an order of magnitude difference. This is probably because in our (HTR) protocol mRNA is directly extracted from the tissue, whereas in IL, rRNA as well as mRNA is concentrated during initial purification, thereby increasing the chance that rRNA will bind non-specifically to the magnetic beads.

### Percentage reads mapped

A similar number of reads mapped with bowtie to the reference gene sets in both HTR and IL library preparation methods and similar number of genes were detected also (Figures 3D,E).

### Coverage analysis

**Figure 4:**
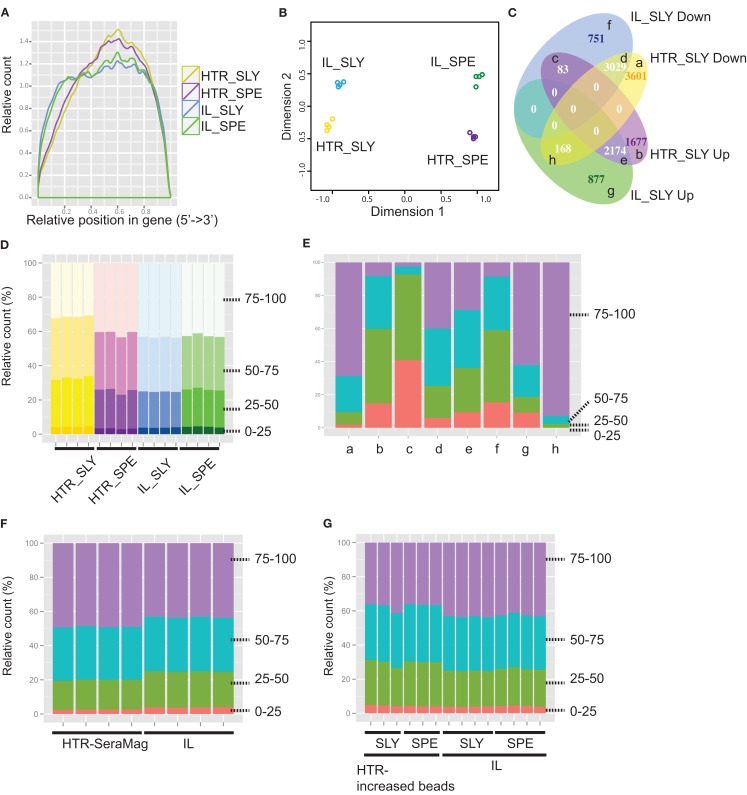
**Detection of gene expression for Illumina (IL) and high-throughput RNA-seq (HTR) library preparations**. **(A)** Read coverage is shown along whole gene length. **(B)** Multidimensional scaling (MDS) plot for assessing the variations amongst samples. Graph was made using the edgeR package in R. **(C)** VennDiagram comparing IL and HTR protocols for differential expressed genes (BH adjusted *p*-value < 0.01) between *S. lycopersicum* (SLY) and *S. pennellii* (SPE). The categories (a–h) are described in Table S5 in Supplementary Material. **(D–G)**: Gene counts by gene length for IL and HTR protocols **(D)**, for each category in (C) **(E)**, for IL and HTR using Sera-Mag beads protocols **(F)**, and for IL and HTR increasing Dynabeads amount protocols **(G)**. 0–25, 25–50, 50–75, and 75–100 are the four gene-length quartiles (the genes separated into quartiles based on percentile gene length). Graphs were made in R using ggplot2.

Libraries prepared with the HTR method showed slightly higher 3′ coverage bias when compared with the Illumina library preps (Figure 4A). This however did not appear to increase variation between replicates. In order to determine which step in the library preparation method caused this difference in coverage, we analyzed the reads from control experiments where we had started with total RNA and proceeded to either fragment the RNA (C1_SLY in Figure S4 in Supplementary Material) or to fragment the cDNA (C2_SLY in Figure S4 in Supplementary Material). The results show that the controls are more similar to IL_SLY than HTR_SLY in coverage. Therefore we can conclude that the coverage bias was not a consequence of cDNA fragmentation, but was rather due to differences in the mRNA isolation techniques between Illumina and HTR methods.

### Differential expression analysis

Prior to differential expression analysis, we assessed the variation between the replicates using a multidimensional scaling (MDS) plot. In the MDS plot, the biological replicates clustered closely, indicating that there is very little variation amongst the replicates and the samples separate by species in the first (main) dimension but also by library preparation methods in the second dimension (Figure 4B). This indicates that besides the species level differences that are expected, there are also protocol specific variations.

We first wanted to see if all samples showed similar distribution of reads across all genes. The histograms of normalized and log2 transformed samples exhibit very similar distributions, indicating that there were no sequencing or library preparation artifacts in any of the libraries (Figure S5 in Supplementary Material). In order to visualize the gene specific differences across the replicates and samples, we have plotted the position of four robustly expressing genes on the histogram, based on their log2 read count values (Figure S5 in Supplementary Material). Although there is variation between the two species, and slightly between the two treatments amongst these genes, very little variation is observed between replicates, indicating that both HTR and IL protocols performed well. This data also shows that the barcodes used in this experiment performed well.

We next asked whether our HTR protocol generated similar expression data to that of the IL protocol. To do this, for each protocol we separately performed a differential expression analysis to determine the number of genes that could be detected as differentially expressed between the two tomato species analyzed, and then compared the two sets from each protocol. We found that an overlapping set of differentially expressed genes was identified by both methods, but that there were subsets of genes that were identified to be differentially expressed only in libraries prepared with one protocol or the other (Figure 4C). In order to see if the differences are also evident in the overall read counts, we extracted the read counts of the genes in each category and averaged over all the replicates for each species/protocol combination after which the average was taken across all genes in that category (Table S5 in Supplementary Material). The differences in average read counts for each category do give support to the observation in Figure 4C. For example, in category (c) SLY down in IL and up in HTR, there are more reads in IL_SPE than IL_SLY, but this is reversed in HTR_SLY and HTR_SPE. Often differential expression analyses can have weak power (and hence false negatives) due to low replication or if the amount of mapped reads is low. Wang et al. (2011b) showed that at least 10 million reads were required to detect most of the genes for differential expression analysis in chicken. In order to see if the variation in differential expression was due to improper detection of low expressed genes (owing to fewer reads sequenced), we redid the differential expression by including genes with higher cutoffs of 5, 10, and 50 reads per million (Figure S6 in Supplementary Material). However, the variant differential expression between the two protocols still persisted, indicating that the variations are not due to low expressed genes. In order to see if some of the non-overlap was indeed due to low replication of reads, we performed two replicate comparisons of the two species between libraries and within libraries (Table S6 in Supplementary Material). An average of 41% of the DE genes detected were found to be unique in one set or the other even when comparing replicates within the same protocol, suggesting that the low power in our analysis could account for many of the uniquely called differential expressed genes that we observed between protocols. One interesting observation to note is that when comparing subsamples taken from the same protocol, there are more common DE genes when using HTR protocol than IL protocol (average 64.27 ± 0.91 and 53.63 ± 1.90%, respectively), and the IL protocol detects more differentially expressed genes overall (Table S6 in Supplementary Material). Both protocols yielded a similar number of reads (Table S4 in Supplementary Material), indicating that there is less technical variation in the HTR samples than IL. This is also reflected in the fact that more genes were classified as being differentially expressed between species when using the HTR protocol instead of the IL protocol when examining all samples (Figure 4C). Thus the HTR protocol appears to increase power to detect differentially expressed genes.

Since there are more unique DE genes in the comparison of sets between protocols, than within protocols, there are still protocol specific variations as suggested by the MDS plot (Figure 4B). The fact that there were some genes detected as upregulated in *S. lycopersicum* in one protocol and downregulated in the other (Figure 4C, “c” and “h”) suggests that these might be a result of artifacts introduced during the library synthesis. Variation in the manner in which longer genes vs. shorter genes are represented in a library could possibly affect the differential expression if the resultant distribution of reads representing different transcript lengths from each of the library methods differs. We therefore decided to compare read counts by gene length for the two protocols.

### Count distribution by gene length

Comparing the number of reads coming from genes of different lengths showed that there is a subset of genes that differ among HTR and IL: HTR showed fewer reads in longer genes compared with IL in *S. lycopersicum* libraries, but not in *S. pennellii* (Figure 4D). In order to determine if this gene-length biased difference in read counts contributed to the variation in differential expression between protocols, we checked the gene counts by gene length for each category of differential expressed genes in Figure 4C. We found that there is a strong bias in the categories showing opposite results among HTR and IL (“c” and “h” in Figure 4E). This suggested that the difference in gene length might be one of main causes of the variation in differential expression among the two protocols. The fact that there is a gene-length bias in *S. lycopersicum* but not in *S. pennellii* suggests that there is likely increased variation between *S. lycopersicum* libraries prepared using different methods than *S. pennellii*. To confirm this idea, we performed a differential expression analysis between the same species but different protocols. This analysis showed more differential expressed genes were detected in the *S. lycopersicum* data set than in the *S. pennellii* data set (7838 genes in *S. lycopersicum* vs. 3324 genes in *S. pennellii*), which strongly suggested that the difference in gene length could cause the conflict in differential expressed gene set among two methods.

To ascertain which specific step led to the gene-length bias, we first compared the C1_SLY and C2_SLY controls but we did not see a gene-length bias in these samples relative to IL (Figure S7 in Supplementary Material), suggesting that cDNA fragmentation and the downstream steps do not create a gene-length bias. We next hypothesized that differences in the way mRNA is purified by the Sera-Mag oligo dT beads in the Illumina protocol and the Dynabeads oligo dT beads in the HTR protocol could account for the gene-length bias. Perhaps owing to the differing binding efficiency, Dynabeads oligo dT beads used in our protocol could have differential affinity for a different subset of longer genes in comparison to Sera-Mag beads. In order to determine if the gene-length bias is due to the direct extraction of mRNA from tissue or due to the choice of beads, we performed the HTR library prep with new *S. lycopersicum* samples, using Sera-Mag oligo dT beads instead of Dynabeads. The read enrichment by gene-length differences was abolished by switching the beads to Sera-Mag (Figure 4F), indicating that the bias might be due to Dynabeads, possibly due to its weaker binding efficiency. We also tested a second solution, increasing the amount of Dynabeads from 25 to 30 μl per sample in HTR to fix the bias in gene length. A reduced bias was observed by just increasing the amount of Dynabeads oligo dT beads (Figure 4G). It is likely that the bias was observed in *S. lycopersicum* samples and not *S. pennellii* was because less tissue was homogenized for *S pennellii* (owing to its smaller leaves), therefore releasing less mRNA. Our *S. lycopersicum* samples likely had saturating amounts of mRNA leading to the observed gene-length biases due to weaker binding efficiency of the Dynabeads. Our results suggest that it is very important to not oversaturate the mRNA isolation beads by excess of tissue lysate or by low bead concentration. In our protocol in the Methods in Supplementary Material, we have provided an option of using either Sera-Mag or Dynabeads oligo dT beads with the recommended bead and sample volumes.

### Library making costs

The library preparation method that we provide here is extremely cost effective. A typical reaction will cost less than USD $27 per sample (from tissue to library) in comparison to Illumina’s mRNA-Seq 8 sample kit ($275 per sample, including RNA extraction and other materials) and Illumina’s TruSeq kit ($90 per sample, including RNA extraction and other materials). Thus, our protocol yields ~3–11× cost reduction from popular available commercial kits, with a cost savings of $6,000–24,000 for 96 samples and $64,000–249,000 for 1000 samples (based on a comparison to TruSeq and mRNA-Seq 8 sample kit, respectively).

## Conclusion

We have successfully developed a cost-effective RNA-Seq protocol that enables rapid processing of multiple samples starting from tissue to finished library. Using this high-throughput protocol, we have successfully isolated and prepared libraries not only from tomato species, *S. lycopersicum* and *S. pennellii*, but also from *Lepidium* sp., *Zea mays*, *Brassica* sp., and even the sea weed: *Caulerpa taxifolia* (Sinha and Maloof Labs, unpublished results). Using our HTR protocol and the new barcoded adapters, we have successfully produced more than 1000 libraries, processing more than 80 samples at a time, and multiplexed to be run in HiSeq sequencers (Sinha and Maloof Labs, unpublished results). We have managed to minimize gene-length-based biases by either switching to Sera-Mag oligo dT beads or increasing the amount of Dynabeads oligo dT beads in our modifications to the protocol. For a given method, coverage biases should be relatively constant for each gene across samples. Therefore the datasets generated using our methods are suitable for gene expression analysis provided samples are not compared with experiments involving different library preparation methods. Various methods have been developed to achieve strand specificity in RNA-Seq and Levin and coworkers recently provided a detailed comparison of several of the techniques (Levin et al., 2010). Of these, the dUTP based method (Parkhomchuk et al., 2009) was found most reliable in that comparison. Although we have not yet tried this, our high-throughput protocol could be easily modified to achieve dUTP method based strand specificity as has been done by Wang et al. (2011a) and Zhong et al. (2011).

## Conflict of Interest Statement

The authors declare that the research was conducted in the absence of any commercial or financial relationships that could be construed as a potential conflict of interest.

## Supplementary Material

The Supplementary Material for this article can be found online at: http://www.frontiersin.org/Plant_Genetics_and_Genomics/10.3389/fpls.2012.00202/abstract

### Presentation 1 includes:

Supplementary Methods S1 **High-throughput RNA-seq (HTR) library preparation**.

Supplementary Methods S2 **Alternative HTR protocol (C1)**.

Supplementary Methods S3 **Alternative HTR protocol (C2)**.

Supplementary Methods S4 **Optimizing cDNA fragmentation with DNA fragmentase; Optimizing size selection using AMPureXP beads by varying PEG concentration**.

Supplementary Methods S5 **Annealing primers to make adapters**.

### Presentation 2 includes:

Supplementary Figure S1 **Overview of Illumina (IL) and high-throughput RNA-seq (HTR) library preparations**.

Supplementary Figure S2 **cDNA fragmentation with DNA fragmentase**.

Supplementary Figure S3 **Size selection using AMPureXP beads by PEG concentration**.

Supplementary Figure S4 **Coverage analysis in C1 and C2 methods**.

Supplementary Figure S5 **Distribution of gene expression levels**.

Supplementary Figure S6 **DE based on higher cutoffs**.

Supplementary Figure S7 **Count distribution by gene length in C1 and C2 methods**.

### Presentation 3 includes:

Supplementary Table S1 **Multiplexing information and read statistics of the samples**.

Supplementary Table S2 **Sequences of 96 five-nt barcoded and 8 three-nt barcoded adapters**.

Supplementary Table S3 **Read statistics of RNA-Seq samples multiplexed with the five-nt barcodes**.

Supplementary Table S4 **Percent reads for each library post processing**.

Supplementary Table S5 **Read counts for various DE categories**.

Supplementary Table S6 **Comparison of differentially expressed gene sets between different groups**.

## Footnotes

^1^http://genomics.xprize.org/

^2^http://tgrc.ucdavis.edu/

^3^http://bioinf.eva.mpg.de/multiplex/

^4^http://www.bioinformatics.bbsrc.ac.uk/projects/fastqc/
