# Supplementary material for: A High-Throughput Method for Illumina RNA-Seq Library Preparation
Source: Front Plant Sci. 2012 Aug 28;3:202. doi: 10.3389/fpls.2012.00202 (PMC3428589; doi:10.3389/fpls.2012.00202)
Supplement: Supplementary Methods S1 — High-throughput RNA-seq (HTR) library preparation. [file 28988_Sinha_Presentation1.PDF]

Supplementary Methods 1
High-throughput RNA-seq (HTR) library preparation

Consumables:

- 2.3 mm Zirconia/Silica beads (Biospec, #11079125z)
- Liquid nitrogen
- 2 ml natural microcentrifuge tubes (ex. USA Scientific Inc, #1620-2700)
- 2 ml vial racks (BioSpec, #702VH45)
- Qiashredder column (Qiagen, #79654)
- 1.2 ml tube racked (ex. USA Scientific Inc, #1412-1400)
- RNase-free 0.2 ml PCR 8-tube strips (ex. USA Scientific Inc, #1402-3500)
- RNase-free 96-well 0.2 ml PCR plate (ex. USA Scientific Inc, #1402-9600)

Devices:

- Beadbeater (Mini Beadbeater 96, BioSpec Products, Inc., USA)
- Magwell 96 well magnetic separator (EdgeBio, #57624)
- MicroPlate Genie™ multiple well plate mixer (USA Scientific Inc, #7400-0400)
- PCR thermocycler

Day 1

1. Direct mRNA purification from tissue using Dynabeads oligo dT magnetic beads

Reagents and enzymes:

- Dynabeads mRNA Direct Kit (Invitrogen, #610-12) or (optional) Sera-Mag oligo dT magnetic beads (Thermo Scientific, Cat. # 3815-2103-010150) (Store at 4°C)

- Buffers:

Make sure you use nuclease-free water if you are making your own solutions. Note that precipitate may form in the buffers. Dissolve precipitate before use by warming to room temperature and mixing thoroughly.

<u>Lysis/Binding Buffer (LBB) (Store at 4°C)</u>

100 mM Tris-HCl, pH 7.5
500 mM LiCl
10 mM EDTA, pH 8
1% LiDS
5 mM DTT
Just before homogenization, add 2-Mercaptoethanol (5 μl/ml) and Antifoam A (15 μl/ml, 0.1-0.2%) and mix thoroughly by vortexing until it is uniformly dispersed.

<u>Washing Buffer A (WBA) (Store at 4°C)</u>

10 mM Tris-HCl, pH 7.5
0.15 M LiCl
1 mM EDTA
0.1% LiDS

<u>Washing Buffer B (WBB) (Store at 4°C)</u>

10 mM Tris-HCl, pH 7.5
0.15 M LiCl
1 mM EDTA

<u>Low-salt Buffer (LSB) (Store at 4°C)</u>

20 mM Tris-HCl, pH 7.5
150 mM NaCl
1 mM EDTA

10 mM Tris-HCl (pH 7.5)(Store at room temperature)

- 10 x DNase I Reaction Buffer (NEB, M0303S) (Store at -20°C)

- DNaseI (NEB, M0303S) (Store at -20°C)

- RNaseOUT (40 U/µl, Invitrogen, #10777-019) (Store at -20°C)
- 25 mM dNTP (Fermentas, #R1121) (Store at -20°C)
- 0.5 M EDTA pH 8.0
- Random Primers (3 µg/µl, Invitrogen, #48190-011) (Store at -20°C)

Procedure:

1.1. Preparation of lysate from plant tissue

1. Add 4-8 2.3 mm Zirconia/Silica beads to 2 ml natural microcentrifuge tubes.

2. Harvest tissue (5-150 mg) into these tubes and immediately freeze in liquid N_2. Transfer to -80°C if storage is necessary.

3. Transfer tube to a pre-chilled 2 ml vial rack, immediately add **1000 µl LBB/100 mg tissue** (add 500 µl to samples that are less than 50 mg), and quickly place in beadbeater.

4. Make sure beadbeater tube rack is balanced and beat for **2 minutes**. Inspect, mix by shaking, and thaw the tissue if required and beat again until tissue well homogenized.

5. Keep at room temperature for at least **5 minutes**.

6. (**Optional step.** Perform this step if the tissue lysate is very viscous) Load the supernatants into a Qiashredder column.

7. Spin for **10 minutes**, max speed.

8. Carefully transfer lysate to new 1.5 ml microcentrifuge tube or into 96-well deep well plates (such as Nunc 2 ml Deep Well U96 PP). Samples can be stored safely at -80°C at this step.

1.2. Bead Preparation

1. Resuspend Dynabeads Oligo(dT) beads thoroughly before use.

2. Transfer the desired volume of beads from the stock tube to a RNase-free tube and place the tube on a magnetic separator (**40-50 µl beads for 200 µl of lysate).** Optional: If using SeraMag beads, use 30-40 µl beads for 200 µl of lysate.

3. When the suspension is clear, remove the supernatant.

4. Wash the beads by resuspending in **100 µl** of LBB buffer (with Antifoam A, 5 µl/ml) and proceed to next mRNA isolation step.

1.3. Direct mRNA Isolation

1. Transfer **100 µl** of beads in LBB per sample (from **step 1.2**) into PCR plate and remove the LBB from the pre-washed beads by placing on a magnetic separator until the suspension is clear, then pipette the liquid off.

2. Add **200 µl** of the sample lysate (from **step 1.1**) to the washed beads (bring the samples to room temperature first if they were stored in -80°C. You can store the remaining lysate back at -80°C).

3. Pipette to resuspend the beads completely in the sample lysate. Incubate mix on a plate mixer for **10 minutes** at room temperature to allow the polyA tail of the mRNA to hybridize to the oligo $(dT)_{25}$ on the beads. Increase the incubation time if the solution is viscous.

4. Place the vial on magnetic separator for 2-10 minutes and remove the supernatant.

5. Wash the beads/mRNA complex with **200 µl** of Washing Buffer A at room temperature. Use the magnetic separator to separate the beads from the solution between each washing step.

6. Wash the beads/mRNA complex with 200 µl volume of Washing Buffer B at room temperature. Make sure the enzyme mix for step 9 is ready before discarding Washing Buffer B and proceeding to next step.

7. Wash with **200 µl** of LSB. Add **16 µl** of 10 mM Tris-HCl, mix and incubate at **80°C** for **2 minutes**. Immediately transfer the tubes to the magnetic separator and transfer the supernatant containing mRNA (should be more than 15 µl) to a fresh PCR tube.

8. Immediately add **100 µl** of WBB to the beads and resuspend.

9. Perform DNase treatment. Make the following (make a pool with 10% more if you have a lot of samples) and mix well:

- 10 x DNase I Reaction Buffer **2.5 µl**
- DNaseI **1 µl**
- RNaseOUT **0.5 µl**

- RNase-free H_2O **6 µl**

<u>The total volume should be **10 µl**</u>

10. Add the mixture to the mRNA sample (**15 µl**, from step 7) and incubate for **10 minutes** at **37°C**.

11. Add **1.5 µl** of 0.5 M EDTA, mix thoroughly and incubate at **75°C** for **10 minutes**.

12. Immediately transfer and keep the sample tubes on ice for **2 minutes**. Add **175 µl** of LBB (with Antifoam A, 15 µl/ml) to the sample and mix well.

13. In the meantime, discard the Washing Buffer B from the beads (from step 8) and wash again with 1**00 µl** of Washing Buffer B.

14. Transfer the sample (from step 12) to the washed beads (from step 13). Incubate at room temperature for **10 minutes**, mixing on a plate mixer.

15. Repeat steps 4, 5, and 6. Make sure the enzyme mix for step 17 is ready before discarding Washing Buffer B and proceeding to the next step.

16. Wash with **200 µl** of LSB.

17. Add following mixture (make a pool with 20% more if you have a lot of samples):

    - 10 mM Tris-HCl **5.8 µl**
    - 25 mM dNTP **0.2 µl**
    - Random Primers mix **0.5 µl**

    <u>The total volume should be **6.5 µl**</u>

18. Incubate at **80°C** for **2 minutes**. Immediately transfer the tubes to the magnetic separator and transfer the supernatant containing mRNA to a fresh PCR plate.

2. First and Second Strand cDNA synthesis

Reagents and enzymes:

- 5 x first strand buffer (Invitrogen, #18080-085) (Store at -20°C)

- 100 mM DTT (Invitrogen, #18080-085) (Store at -20°C)
- SuperScript III (200 U/µl, Invitrogen, #18080-085) (Store at -20°C)
- RNaseOUT (40 U/µl, Invitrogen, #10777-019) (Store at -20°C)
- 10 x DNA Pol rxn buffer (Fermentas, #EP0042) (Store at -20°C)
- DNA Pol I (10 U/µl, Fermentas, #EP0042) (Store at -20°C)
- 25 mM dNTP (Fermentas, #R1121) (Store at -20°C)
- RNaseH (5 U/µl, NEB, #M0297S) (Store at -20°C)
- Ampure XP beads (Beckman, #A63881) (Store at 4°C)
- Ampure XP Bead Resuspension Buffer (ABR) (Store at room temperature)

15% PEG 8000
2.5 M NaCl

- 75% ethanol (Store at -20°C)
- 10 mM Tris (pH 7.5)(Store at room temperature)

Procedure:

2.1. First strand cDNA synthesis

1. Incubate the sample in a PCR thermocycler at **65°C** for **5 minutes**, and then place the tube on ice.
2. Make the following (make a pool with 20% more if you have a lot of samples) and mix well:
    - 5 x first strand buffer **2 µl**
    - 100 mM DTT **0.5 µl**
    - RNaseOUT **0.5 µl**
    - SuperScript III **0.5 µl**

    The total volume should be **3.5 µl**
3. Add the mixture to the sample and mix well.

4. Incubate the sample in a PCR thermocycler with following program:

    - 25°C 10 minutes
    - 50°C 60 minutes
    - 70°C 15 minutes
    - 4°C Hold

5. Place tubes on ice.

2.2. Second strand cDNA synthesis

1. Make the following (make a pool with 10% more if you have a lot of samples) and mix well:

- 10 x DNA Pol rxn buffer **5 µl**
- 25 mM dNTP **0.6 µl**
- RNase-free H_2O **31.7 µl**

<u>The total volume should be **37.3 µl**</u>

2. Add the mixture to the sample and incubate on ice until well chilled.

3. Make the following (make a pool with 20% more if you have a lot of samples) and mix well:

- RNaseH **0.2 µl**
- DNA Pol I **2.5 µl**

<u>The total volume should be **2.7 µl**</u>

4. Add the mixture to the sample and incubate in a PCR thermocycler at **16°C** for **2.5 hours**.

2.3. Bead purification

1. Add **30 µl** Ampure XP beads and **49 µl** ABR to the sample and mix. Keep at room temperature for **5 minutes**.

2. Place the samples on the magnetic separator and remove the supernatant once the beads are pulled towards the magnet and the solution is clear.

3. Wash with **200 µl** of 75% ethanol (it is not necessary to dislodge the magnetic pellet but make sure the tube is thoroughly rinsed with the ethanol). Remove ethanol without disturbing the bead pellet and let it dry for **2-5 minutes**.

4. Elute in **7 µl** of 10 mM Tris-HCl. You can quantify up to 2 µl of the cDNA at this point. It is not necessary once you have determined that the protocol works well for your species and tissue. Proceed to day 2 if you have at least 20 ng/µl of cDNA)

Day 2

3. cDNA fragmentation and bead purification

Reagents and enzymes:

- 10 x Fragmentation Buffer (NEB, #M0348L) (Store at -20°C)
- 10 x BSA (NEB, #M0348L) (Store at -20°C) The Fragmentase comes with 100 x BSA. Make a 10 x BSA stock out of this.
- Fragmentase (NEB, #M0348L) (Store at -20°C)
- 500 mM EDTA (Store at room temperature)
- 10 mM Tris (pH 7.5)(Store at room temperature)
- Ampure XP beads (Beckman, #A63881) (Store at 4°C)
- 75% ethanol (Store at -20°C)

Procedure:

1. Transfer **4 µl** (or up to 150 ng) of cDNA to a fresh PCR plate, and keep on ice.

2. Make the following (make a pool with 20% more if you have a lot of samples), mix well and chill on ice until cold:

- 10 x Fragmentation Buffer **1 µl**
- 10 x BSA **1 µl**
- RNase-free H_2O **3 µl**
- Fragmentase **1 µl**

The total volume should be **6 µl**

3. Add the mixture to the sample, mix well and incubate in a PCR thermocycler at **37°C** for **30 minutes**, and then keep at **4°C**.

4. Prepare a mix of **2 µl** of 500 mM EDTA and **8 µl** of 10 mM Tris-HCl and add to the sample.

5. Add **30 µl** Ampure XP beads and mix. Keep at room temperature for **5 minutes**.

6. Place the samples on the magnetic separator and remove the supernatant once the beads are pulled towards the magnet and the solution is clear.

7. Wash with **200 µl** of 75% ethanol (it is not necessary to dislodge the magnetic pellet but make sure the tube is thoroughly rinsed with the ethanol). Remove ethanol without disturbing the bead pellet.

8. Let it dry for **2-5 minutes** and immediately proceed to next step.

4. End repair, A-tailing and adapter ligation

Reagents and enzymes:

- 10 x NEBNext End repair buffer (NEB, #E6050L) (Store at -20°C)
- NEBNext End repair enzyme mix (NEB, #E6050L) (Store at -20°C)
- Klenow DNA polymerase (5 U/µl, NEB, #M0210S) (Store at -20°C)
- E.coli DNA ligase (NEB, #M0348L) (Store at -20°C)
- ABR (see **section 2**)
- 75% ethanol (Store at -20°C)
- 10 x Klenow buffer (Fermentas, #EP0422)

- Klenow 3’ to 5’ exo- (5 U/µl, Fermentas, #EP0422)
- 100 mM dATP (Fermentas, #R0141) Make a 1 mM dATP stock out of this 100 mM dATP.
- Adapter oligo mix (10 ng/µl stock; 50 ng total) (Store at -20°C)
- 2 x Rapid T4 DNA Ligase Buffer (Enzymatics, #L603-HC-L) (Store at -20°C)
- DNA Ligase (600 U/µl, Enzymatics, #L603-HC-L) (Store at -20°C)
- 10 mM Tris (pH 7.5) (Store at room temperature)

Procedure:

4.1. End repair

1. Make the following (make a pool with 10% more if you have a lot of samples) and mix well:

    - RNase-free H_2O **20.75 µl**
    - 10 x NEBNext End repair buffer **2.5 µl** (make sure ATP/salts in the buffer are completely dissolved)
    - NEBNext End repair enzyme mix **1.25 µl**
    - Klenow DNA polymerase **0.25 µl**
    - E.coli DNA ligase (from NEB cDNA fragmentase kit) **0.25 µl**

    <u>The total volume should be **25 µl**</u>

2. Add the mixture to the sample, and incubate in a PCR thermocycler at **20°C** for **30 minutes**.

3. Add **40 µl** ABR and mix. Keep at room temperature for **5 minutes**.

4. Place the samples on the magnetic separator and remove the supernatant once the beads are pulled towards the magnet and the solution is clear.

5. Wash with **200 µl** of 75% ethanol (it is not necessary to dislodge the magnetic pellet but make sure the tube is thoroughly rinsed with the ethanol). Remove ethanol without disturbing the bead pellet.

6. Let it dry for **2-5 minutes** and immediately proceed to next step.

4.2. A-tailing

1. Make the following (make a pool with 10% more if you have a lot of samples) and mix well:

- RNase-free H_2O **8 µl**
- 10 x Klenow buffer **1.25 µl**
- 1 mM dATP **2.5 µl**
- Klenow 3' to 5' exo- **0.75 µl**

The total volume should be **12.5 µl**

2. Add the mixture to the sample, and incubate in a PCR thermocycler at **37°C** for **30 minutes**.

3. Increase the volume to 20 µl by adding **7.5 µl** of 10 mM Tris-HCl.

4. Add **32 µl** ABR and mix. Keep at room temperature for **5 minutes**.

5. Place the samples on the magnetic separator and remove the supernatant once the beads are pulled towards the magnet and the solution is clear.

6. Wash with **200 µl** of 75% ethanol (it is not necessary to dislodge the magnetic pellet but make sure the tube is thoroughly rinsed with the ethanol). Remove ethanol without disturbing the bead pellet.

7. Let it dry for **2-5 minutes** and immediately proceed to next step.

4.3. Adapter ligation

1. Add **5 µl** of Adapter oligo mix (10 ng/µl stock; 50 ng total) to the sample.

2. Make the following (make a pool with 20% more if you have a lot of samples) and mix well:

- RNase-free H_2O **1.5 µl**
- 2 x Rapid T4 DNA Ligase Buffer **6.75 µl**
- DNA Ligase **0.25 µl**

The total volume should be **8.5 µl**

3. Add the mixture to the sample, and incubate at room temperature for **15 minutes**.

4. Increase the volume to 20 µl by adding **6.5 µl** of 10 mM Tris-HCl.

5. Add **15 µl** ABR and mix. Keep at room temperature for **5 minutes**.

6. Place the samples on the magnetic separator and remove the supernatant once the beads are pulled towards the magnet and the solution is clear.

7. Wash with **200 µl** of 75% ethanol (it is not necessary to dislodge the magnetic pellet but make sure the tube is thoroughly rinsed with the ethanol). Remove ethanol without disturbing the bead pellet.

8. Let it dry for **2-5 minutes** and immediately proceed to next step.

9. Elute in **30 µl** of 10 mM Tris-HCl.

5. PCR Enrichment

Reagents and enzymes:

- 5 x Phusion Buffer (NEB, #M0530S) (Store at -20°C)
- Phusion polymerase (NEB, #M0530S) (Store at -20°C)
- 10 µM PCR primer PE 1.0 (Store at -20°C; 5’AATGATACGGCGACCACCGAGATCTACACTCTTTCCCTACACGACGCTCTTCCGATCT3’)
- 10 µM PCR primer PE 2.0 (Store at -20°C; 5’CAAGCAGAAGACGGCATACGAGATCGGTCTCGGCATTCCTGCTGAACCGCTCTTCCGATCT3’)
- 25 mM dNTP (Fermentas, #R1121) (Store at -20°C)
- Ampure XP beads (Beckman, #A63881) (Store at 4°C)
- 75% ethanol (Store at -20°C)
- 10 mM Tris (pH 7.5) (Store at room temperature)

Procedure:

1. Make the following (make a pool with 10% more if you have a lot of samples) and mix well:

- 5 x Phusion Buffer **5 µl**
- 10 µM PCR primer PE 1.0 **1 µl**
- 10 µM PCR primer PE 2.0 **1 µl**
- 25 mM dNTP **0.25 µl**
- Phusion polymerase **0.25 µl**
- RNase-free H_2O **2.5 µl**

The total volume should be **10 µl**

2. Transfer **15 µl** of the sample to a new tube and add **10 µl** of the reaction mix.

3. Incubate the sample in a PCR thermocycler with following program:

- 98°C 30 seconds
- 98°C 10 seconds ⎫
- 65°C 30 seconds ⎬ 10 x
- 72°C 30 seconds ⎭
- 72°C 5 minutes
- 4°C Hold

4. (Optional step) At this point, you may take out **2 µl** of the library and run on a gel to see if the enrichment is of the desired size to assess the extent of adapter contamination.

5. Add **20 µl** Ampure XP beads and mix. Keep at room temperature for **5 minutes**.

6. Wash **twice** with 75% ethanol (**200 µl**) and then remove ethanol. Let it dry for **5 minutes**.

7. Elute in **15 µl** of 10 mM Tris-HCl.

8. Check **2 µl** on a gel to see if the enrichment is of the desired size and if there is no visible adapter contamination.

Ordering Information:

Company	Qty	Cat#	Name	Quantity	Price ($/ea)	Total price
Biospec	1	11079125z	2.3 mm Zirconia/Silica beads	454 g	50	50
Fermentas	1	R1121	25 mM dNTP	1 ml	190	190
	4	EP0042	DNA Pol I	2500 units (10 units/µl)=250 µl	278	1112
	1	EP0422	Klenow 3' to 5' exo- (5 units/µl)	1500 units (5 units/µl)=300 µl	363	363
	1	R0141	100 mM dATP	250 µl	55	55
Invitrogen	1	610-12	Dynabeads mRNA Direct Kit	10 ml	672	672
	3	10777-019	RNaseOUT™ Recombinant Ribonuclease Inhibitor 40 units/µl	5000 units = 125 µl	140	420
	2	48190-011	Random Primers	9 units: 100 µl	138	276
	1	18080-085	SuperScript™ III Reverse Transcriptase	4 × 10,000 units (SSIII 50 µl x 4)	970	970
NEB	1	M0303S	DNase I (RNase-free) 2,000 units/ml	1000 units	63	63
	2	M0297S	RNase H 5,000 units/ml	250 units	61	122
	1	M0348L	NEBNext™ dsDNA Fragmentase™	250 reactions = 500 µl	95	95
	1	E6050L	NEBNext® End Repair Module	100 reactions: mix 0.5 ml	340	340
	3	M0210S	DNA Polymerase I, Large (Klenow) Fragment 5,000 units/ml	200 units: 40 µl	56	168
	2	M0530S	Phusion® High-Fidelity DNA Polymerase 2,000 units/ml	100 units: 50 µl	103	206
Beckman	1	A63881	AMPure XP	60 ml	1000	1000

Enzymatics	1	L603-HC-L	T4 DNA Ligase (Rapid) 600 units/µl	240000 units: 400 µl	350	350
QIAGEN	2	79656	QIAshredder (250)		301	602
Sigma	1	L7026-500ml	Lithium chloride solution (8 M)	500 ml	63	63
	1	89510-250G-F	Poly(ethylene glycol) BioUltra 8,000	250 g	29.1	29.1
	1	L4632-5G	Lithium dodecly sulfate	5 g	34.4	34.4

These prices cover enough volume for 320 samples + 10% extra, but does not include tips, tubes, other equipment.

Total = less than $ 22.4 per sample

Supplementary Methods 2

Alternative HTR protocol (C1)

This protocol starts with total RNA and has a mRNA fragmentation step instead of the cDNA fragmentation step.

1. mRNA purification

Reagents and enzymes:

- Bead binding buffer

  20 mM Tris-HCl, pH 7.5
  1.0 M LiCl
  2 mM EDTA
  0.2% Tween 20

- Bead washing buffer

  10 mM Tris-HCl, pH 7.5
  0.15 M LiCl
  1 mM EDTA
  0.1% Tween 20

- Sera-Mag oligo dT magnetic beads (Thermo Scientific, Cat. # 3815-2103-010150) (Store at 4°C)
- 10 mM Tris (pH 7.5) (Store at room temperature)
- Nuclease-free water (Store at room temperature)

Procedure:

1. Dilute the total RNA (**5 µg**) with nuclease free water to **25 µl**.

2. Incubate the sample at **65°C** for **10 minutes** to disrupt the secondary structures and then place the tube on ice.

3. Re-suspend Sera-Mag oligo (dT) beads thoroughly before use.

4. Transfer the desired volume of beads from the stock tube to a RNase-free tube and place the tube on a magnetic separator (**7.5 µl each sample**).

5. When the suspension is clear, remove the supernatant.

6. Wash the beads **twice** by re-suspending in **50 µl** of bead binding buffer and remove the supernatant.

7. Re-suspend the beads in **25 µl** of bead binding buffer and add the **25 µl** of total RNA sample from **step 2**.

8. Keep the tube at room temperature for **5 minutes** and remove the supernatant. Use the magnetic separator to separate the beads from the solution between each washing step.

9. Aliquot **25 µl** of bead binding buffer to a fresh Rnase-free tube.

10. Wash the beads/mRNA complex (from **step 8**) **twice** with the **100 µl** of bead washing buffer at room temperature and remove the supernatant.

11. Add **25 µl** of 10 mM Tris-HCl to the beads and then Incubate the sample at **80°C** for **2 minutes** to elute the mRNA from the beads. Immediately transfer the tubes to the magnetic separator and transfer the supernatant containing mRNA to the tube from **step 9**.

12. Immediately add **100 µl** of bead washing buffer to the beads and re-suspend.

13. Incubate the sample at **65°C** for **10 minutes** to disrupt the secondary structures and then place the tube on ice.

14. Wash the beads (from **step 12**) **twice** with the **100 µl** of bead washing buffer at room temperature and remove the supernatant.

15. Transfer the mRNA sample (should be 50 µl, from step 13) to the washed beads (from step 14).

16. Rotate the tube at room temperature for **5 minutes** and remove the supernatant.

17. Wash the beads/mRNA complex **twice** with the **100 µl** of bead washing buffer at room temperature and remove the supernatant.

18. Add **9.5 µl** of 10 mM Tris-HCl to the beads and then incubate the sample at **80°C** for **2 minutes** to elute the mRNA from the beads. Immediately transfer the tubes to the magnetic separator and transfer the supernatant containing mRNA to a fresh RNase-free tube. The resulting volume of mRNA should be approximately 9 µl.

2. RNA fragmentation

Reagents and enzymes:

- Ambion RNA fragmentation buffer and Stop solution (Amvion, Cat. # AM8740) (Store at room temperature)
- 3 M NaOAC (Store at 4°C)
- Glycogen (Store at -20°C)
- 100% and 75% ethanol (Store at -20°C)
- Nuclease-free water (Store at room temperature)

Procedure:

1. Prepare the following reaction mix in a RNase-free 0.2 ml PCR 8-tube strips:
   - 10 x Fragmentation Buffer **1 µl**
   - mRNA **9 µl**

   <u>The total volume should be **10 µl**</u>

2. Set the PCR thermocycler to **70°C** before putting the tube in to avoid ramping time. Incubate in a PCR thermocycler at **70°C** for exactly **5 minutes**.

3. Add **1 µl** of Stop solution, and then place the tube on ice.

4. Transfer the solution to a 1.5 ml microcentrifuge tube. Add the following to the tube and incubate **-80°C** for **30 minutes**:

    - 3 M NaOAC **1 µl**
    - Glycogen **2 µl**
    - 100% ethanol **30 µl**

5. Centrifuge the tube at **14,000 rpm** for **25 minutes** at **4°C** in a microcentrifuge.
6. Carefully remove the supernatant without dislodging the RNA pellet.
7. Wash the pellet with **150 µl** of 75% ethanol.
8. Centrifuge the tube at **14,000 rpm** for **2 minutes** at **4°C** in a microcentrifuge.
9. Carefully remove the supernatant without dislodging the RNA pellet.
10. Let it dry for **10 minutes**.
11. Re-suspend the RNA in **5.6 µl** of nuclease-free water.

3. First and Second Strand cDNA synthesis

Reagents and enzymes:

- Random Primers (3 µg/µl, Invitrogen, #48190-011) (Store in -20°C)
- 5 x first strand buffer (Invitrogen, #18080-085) (Store at -20°C)
- 100 mM DTT (Invitrogen, #18080-085) (Store at -20°C)
- 25 mM dNTP (Fermentas, #R1121) (Store at -20°C)
- SuperScript II (200 U/ µl, Invitrogen, #18064-014) (Store at -20°C)
- RNaseOUT (40 U/µl, Invitrogen, #10777-019) (Store at -20°C)
- 10 x DNA Pol rxn buffer (Fermentas, #EP0042) (Store at -20°C)
- DNA Pol I (10 U/µl, Fermentas, #EP0042) (Store at -20°C)

- RNaseH (5 U/µl, NEB, #M0297S) (Store at -20°C)
- Ampure XP beads (Beckman, #A63881) (Store at 4°C)
- Ampure XP Bead Resuspension Buffer (ABR) (Store at room temperature):

  15% PEG 8000
  2.5 M NaCl

- 75% Ethanol (Store at -20°C)
- 10 mM Tris (pH 7.5)(Store at room temperature)

Procedure:

3-1. First strand cDNA synthesis

1. Prepare the following reaction mix in a RNase-free 0.2 ml PCR 8-tube strips:

   - Random primers **0.5 µl**
   - mRNA **5.55 µl**

   <u>The total volume should be **6.05 µl**</u>

2. Incubate the sample in a PCR thermocycler at **65°C** for **5 minutes**, and then place the tube on ice.

3. Make the following (make a pool with 20% more if you have a lot of samples) and mix well:

   - 5 x first strand buffer **2 µl**
   - 100 mM DTT **1 µl**
   - 25 mM dNTP **0.2 µl**
   - RNaseOUT **0.25 µl**

   <u>The total volume should be **3.45 µl**</u>

4. Add the mixture to the sample and mix well.

5. Incubate the sample in a PCR thermocycler at **25°C** for **2 minutes**.

6. Add **0.5 µl** of SuperScript II to the sample, and incubate the sample in a PCR thermocycler with following program:

    - 25°C 10 minutes
    - 42°C 50 minutes
    - 70°C 15 minutes
    - 4°C Hold

7. Place tubes on ice.

3-2. Second strand cDNA synthesis

1. Make the following (make a pool with 10% more if you have a lot of samples) and mix well:

    - 10 x DNA Pol rxn buffer **5 µl**
    - 25 mM dNTP **0.6 µl**
    - Rnase free H_2O **31.7 µl**

    The total volume should be **37.3 µl**

2. Add the mixture to the sample and incubate on ice until well chilled.

3. Mix the following (make a pool with 20% more if you have a lot of samples) and mix well:

    - RNaseH **0.2 µl**
    - DNA Pol I **2.5 µl**

    The total volume should be **2.7 µl**

4. Add the mixture to the sample and incubate in a PCR thermocycler at **16°C** for **2.5 hours**.

3-3. Bead purification

1. Add **30 µl** Ampure XP beads and **49 µl** ABR to the sample and mix. Keep at room temperature for **5 minutes**.

2. Wash with 75% ethanol (**200 µl**) and then remove ethanol. Let it dry for **2 minutes**.

3. Let it dry for **5 minutes** and immediately proceed to next step.

4. End repair, A-tailing and adapter ligation with-beads

See original HTR protocol.

5. PCR Enrichment

See original HTR protocol.

Supplementary Methods 3

Alternative HTR protocol (C2)

This protocol starts with total RNA.

1. mRNA purification

Follow the same procedure in Alternative HTR protocol (C1) except for step 18. Instead, follow the steps below.

1. Add following mixture (make a pool with 20% more if you have a lot of samples):

    - 10 mM Tris-HCl **5.8 µl**
    - 25 mM dNTP **0.2 µl**
    - Random Primers mix **0.5 µl**

    The total volume should be **6.5 µl**

2. Incubate at **80°C** for **2 minutes**. Immediately transfer the tubes to the magnetic separator and transfer the supernatant containing mRNA to a fresh PCR plate.

2. First and Second Strand cDNA synthesis

See original HTR protocol.

3. cDNA Fragmentation and bead purification

See original HTR protocol.

4. End repair, A-tailing and adapter ligation with-beads

See original HTR protocol.

5. PCR Enrichment

See original HTR protocol.

Supplementary Methods 4

Optimizing cDNA fragmentation with DNA fragmentase

This was performed to determine the proper incubation time for DNA fragmentation by NEBNext® DNA fragmentase enzyme mix (NEB, Beverley, MA) in the HTR protocol. 100 bp DNA ladder (Fermentas, Cat. # SM1143) was first purified using the QIAquick PCR Purification kit (Qiagen, Cat. # 28104). 500 ng and 100 ng of the purified DNA ladder were then digested with DNA fragmentase enzyme mix for different incubation times (from 0 to 45 minutes) according to the manufacturer's protocol. The fragmentation of different DNA quantities (500 ng and 100 ng) showed similar patterns, indicating that fragmentation by the DNA fragmentase enzyme mix is less dependent on the DNA quantity, at least for the range tested. For our library preparations we aimed for an optimal range of 200-300 bp (which after addition of adapters becomes 300-400 bp), and this was best obtained after 30 minutes of fragmentation (Supplementary Figure 2).

Optimizing size selection using AMPureXP beads by varying PEG concentration

This was performed to determine the optimal PEG concentration for purification by AMPureXP beads. 100 bp DNA ladder (Fermentas, Cat. # SM1143) was first purified using the QIAquick PCR Purification kit (Qiagen, Cat. # 28104). 150 ng of this DNA ladder was then purified with a constant volume of AMPure XP beads (Beckman, Cat. # A63881; corresponding 20 μl each sample of original AMPure XP beads), but with different PEG and NaCl concentrations (Supplementary Figure 3). The cleanup procedure was followed according to the manufacturer's protocol.

Supplementary Methods 5

Annealing primers to make adapters

Reagents and enzymes:

- 10 x annealing buffer

  100 mM Tris-HCl, pH 7.5

  500 mM NaCl

  10 mM EDTA

- PE1/PE2 barcoded primers

Procedure:

1. Add following mixture for each adapter:

   - 10 x annealing buffer **1 µl**
   - PE1 barcoded primer **4.5 µl**
   - PE2 barcoded primer **4.5 µl**

   The total volume should be **10 µl**

2. Incubate the mixture in a PCR thermocycler with following program:

   - 95°C 2.5 minutes
   - 95°C 30 seconds 70 cycles (-1°C per cycle)
   - 25°C 10 minutes
   - 4 °C Hold

3. Quantify the products and dilute by adding water to 10 ng/µl.

4. Store at -20°C.
