# Supplementary material for: A High-Throughput Method for Illumina RNA-Seq Library Preparation
Source: Front Plant Sci. 2012 Aug 28;3:202. doi: 10.3389/fpls.2012.00202 (PMC3428589; doi:10.3389/fpls.2012.00202)

Supplementary Figure 1

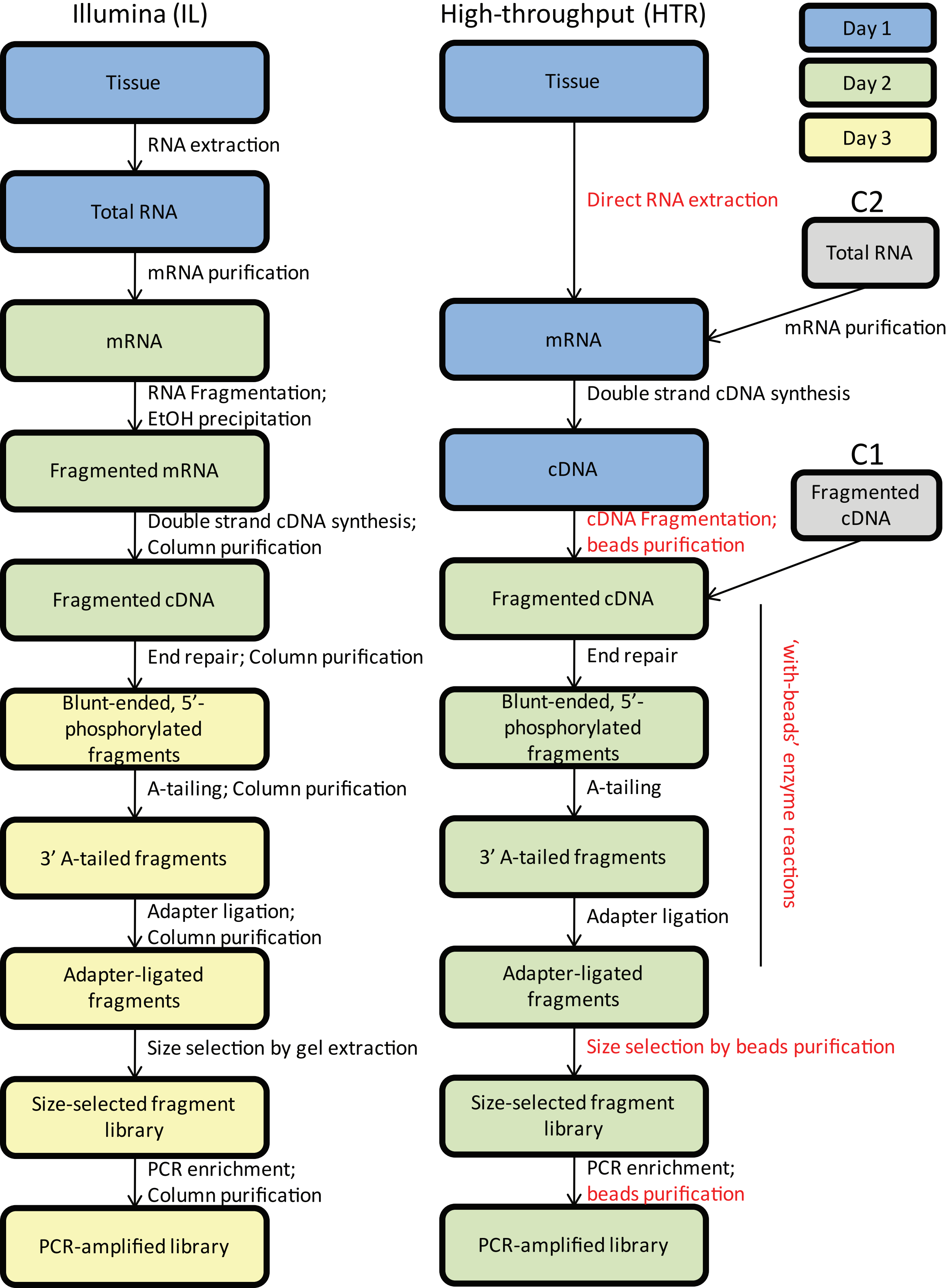

Supplementary Figure 2

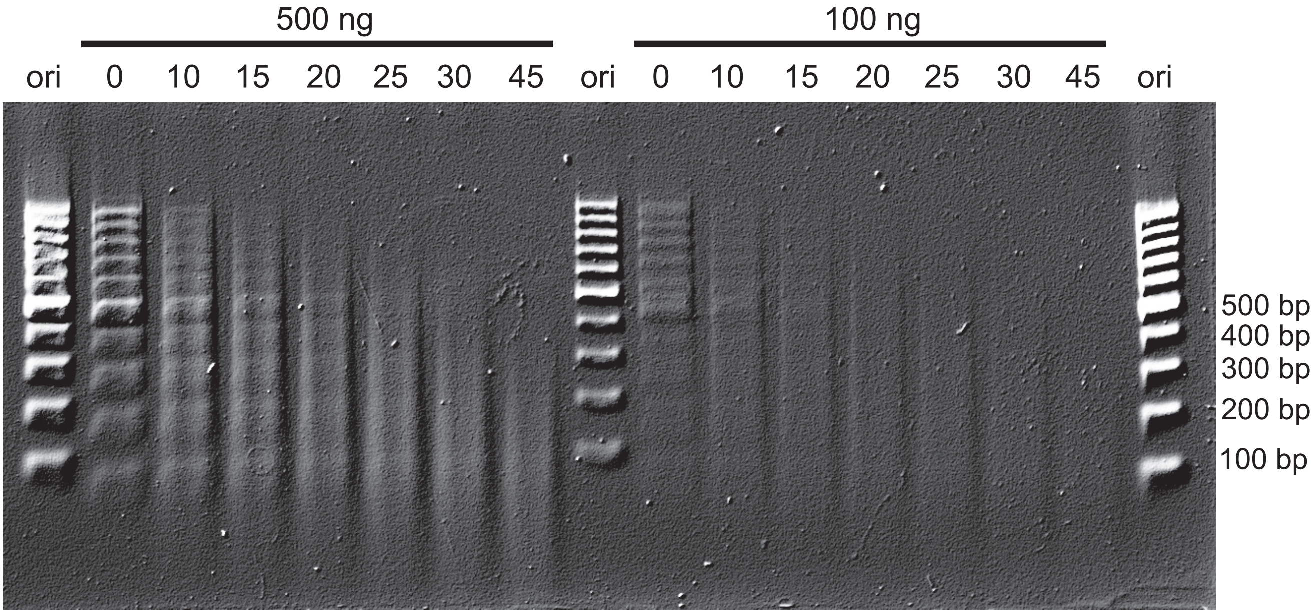

Supplementary Figure 3

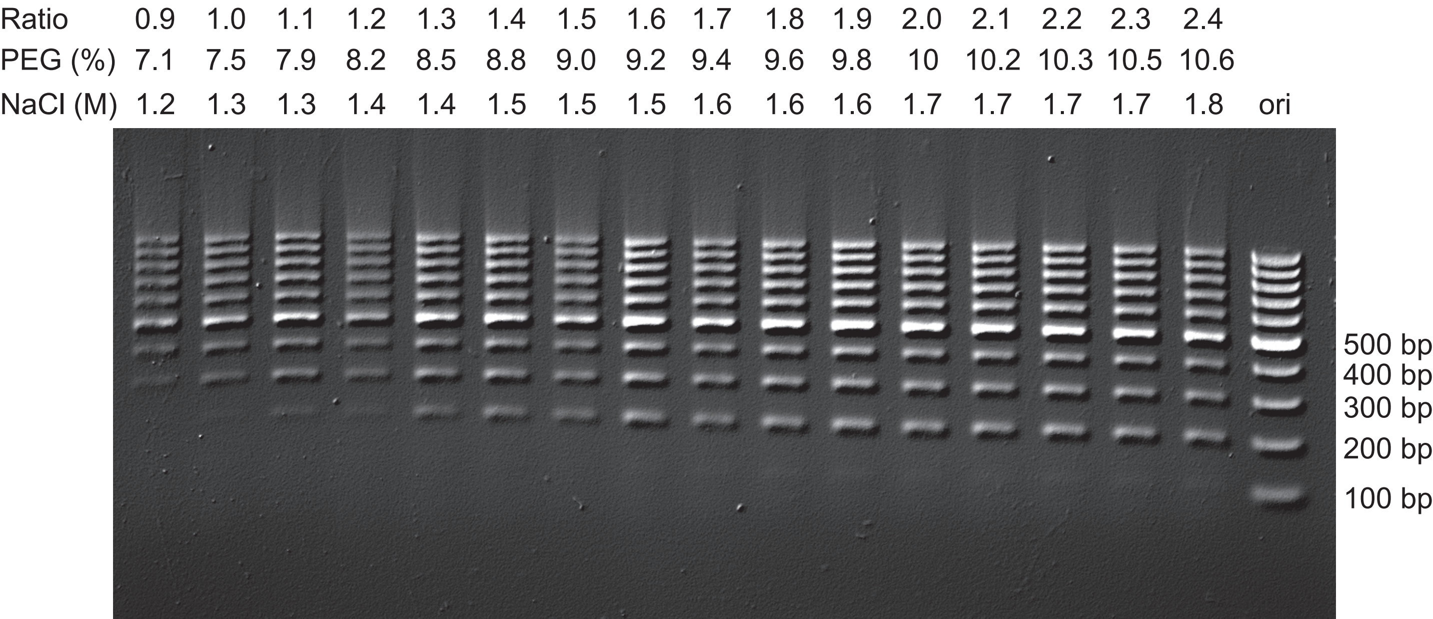

Supplementary Figure 4

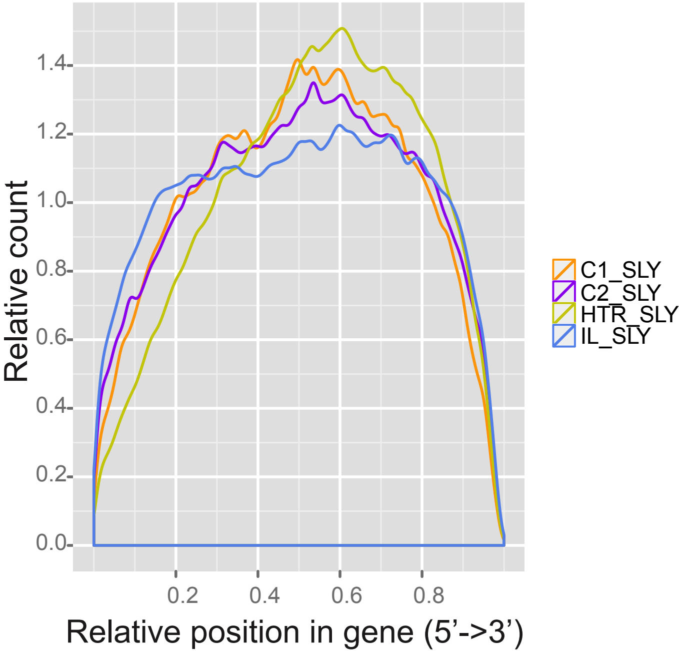

Supplementary Figure 5

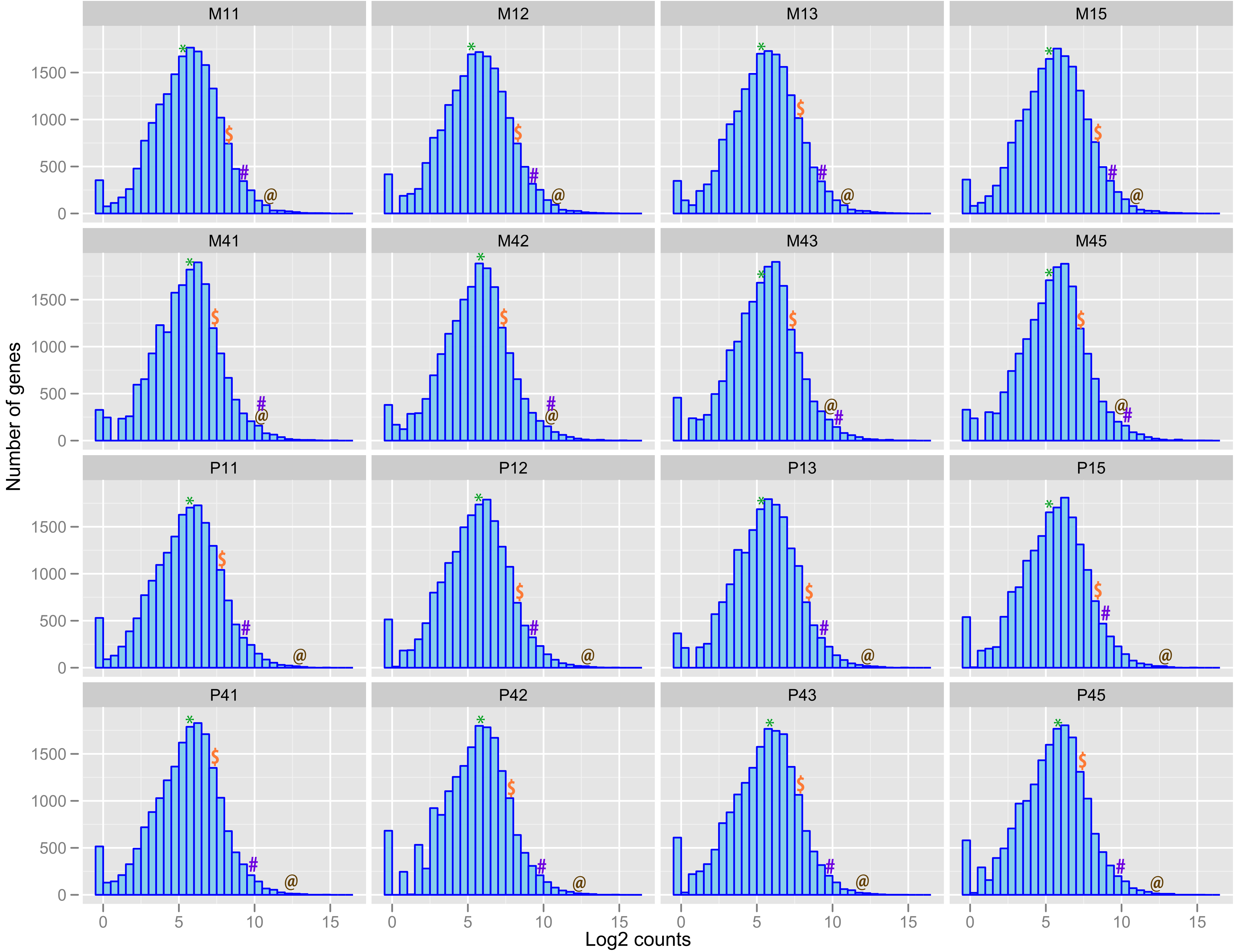

Supplementary Figure 6

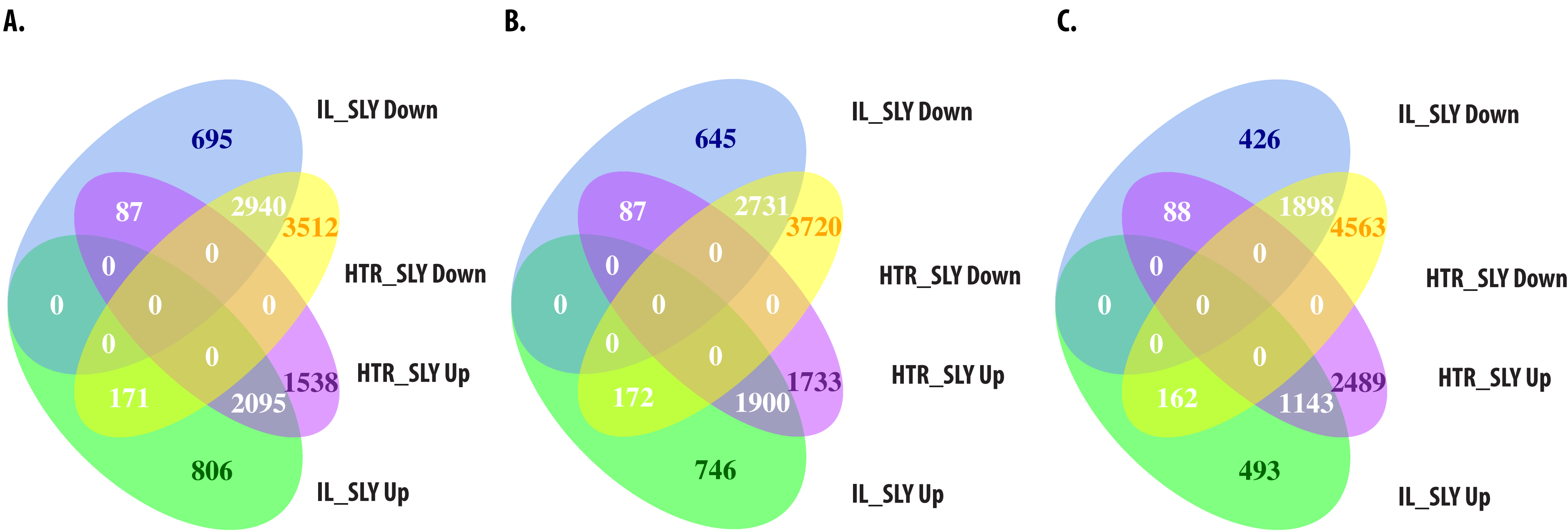

Supplementary Figure 7

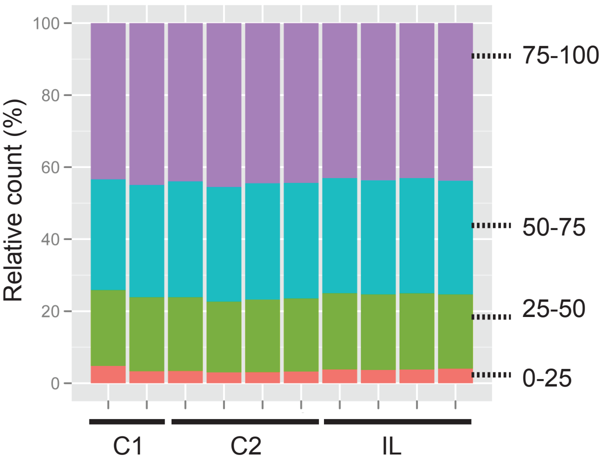

Supplement: Supplementary Figure S1 — Overview of Illumina (IL) and high-throughput RNA-seq (HTR) library preparations. [file 28988_Sinha_Presentation2.PDF]
