# Supplementary material for: A High-Throughput Method for Illumina RNA-Seq Library Preparation
Source: Front Plant Sci. 2012 Aug 28;3:202. doi: 10.3389/fpls.2012.00202 (PMC3428589; doi:10.3389/fpls.2012.00202)
Supplement: Supplementary Table S1 — Multiplexing information and read statistics of the samples. [file 28988_Sinha_Presentation3.PDF]

Supplementary Table 1

Sample	Barcode	Pool	Total reads	Percent mapped
P12	AGG	HTR_1_8	4499977	72.21
P42	CAC	HTR_1_8	3564549	74.89
M23	GCT	HTR_1_8	2553120	72.73
P11	TTG	HTR_1_8	4810008	73.81
P43	ACC	HTR_1_8	4004353	75.16
M42	CTA	HTR_1_8	3099471	74.74
M41	GGA	HTR_1_8	3635645	75.28
M11	TAT	HTR_1_8	6253353	77.06
M43	AGG	HTR_9_16	4355405	72.65
P15	CAC	HTR_9_16	4186029	73.43
M33	GCT	HTR_9_16	5216050	65.46
M22	TTG	HTR_9_16	*	NA
M25	ACC	HTR_9_16	4278895	74.92
M15	CTA	HTR_9_16	5936521	75.56
P41	GGA	HTR_9_16	5571477	72.23
M32	TAT	HTR_9_16	6362214	64.11
M21	AGG	HTR_17_24	*	NA
M45	CAC	HTR_17_24	3435645	75.28
P45	GCT	HTR_17_24	2276871	73.63
P13	TTG	HTR_17_24	3517571	72.52
M31	ACC	HTR_17_24	1508380	64.12
M12	CTA	HTR_17_24	4199127	76.70
M13	GGA	HTR_17_24	3008295	76.22
M35	TAT	HTR_17_24	4106501	63.37

Supplementary Table 2

Oligonucleotides for adatpers with 5 nucleotide barcodes (96 in total)

Forward	Sequence	Reverse	Sequence2
PE1-AAGAC	P-GTCTTAGATCGGAAGAGCGGTTCAGCAGGAATGCCGAG	PE2-AAGAC	ACACTCTTTCCCTACACGACGCTCTTCCGATCTAAGACT
PE1-ACCAT	P-ATGGTAGATCGGAAGAGCGGTTCAGCAGGAATGCCGAG	PE2-ACCAT	ACACTCTTTCCCTACACGACGCTCTTCCGATCTACCATT
PE1-AGACG	P-CGTCTAGATCGGAAGAGCGGTTCAGCAGGAATGCCGAG	PE2-AGACG	ACACTCTTTCCCTACACGACGCTCTTCCGATCTAGACGT
PE1-CATTA	P-TAATGAGATCGGAAGAGCGGTTCAGCAGGAATGCCGAG	PE2-CATTA	ACACTCTTTCCCTACACGACGCTCTTCCGATCTCATTAT
PE1-GCTGC	P-GCAGCAGATCGGAAGAGCGGTTCAGCAGGAATGCCGAG	PE2-GCTGC	ACACTCTTTCCCTACACGACGCTCTTCCGATCTGCTGCT
PE1-GTAGG	P-CCTACAGATCGGAAGAGCGGTTCAGCAGGAATGCCGAG	PE2-GTAGG	ACACTCTTTCCCTACACGACGCTCTTCCGATCTGTAGGT
PE1-TGCCT	P-AGGCAAGATCGGAAGAGCGGTTCAGCAGGAATGCCGAG	PE2-TGCCT	ACACTCTTTCCCTACACGACGCTCTTCCGATCTTGCCTT
PE1-TGGTA	P-TACCAAGATCGGAAGAGCGGTTCAGCAGGAATGCCGAG	PE2-TGGTA	ACACTCTTTCCCTACACGACGCTCTTCCGATCTTGGTAT
PE1-AACCG	P-CGGTTAGATCGGAAGAGCGGTTCAGCAGGAATGCCGAG	PE2-AACCG	ACACTCTTTCCCTACACGACGCTCTTCCGATCTAACCGT
PE1-ATTGA	P-TCAATAGATCGGAAGAGCGGTTCAGCAGGAATGCCGAG	PE2-ATTGA	ACACTCTTTCCCTACACGACGCTCTTCCGATCTATTGAT
PE1-CAACT	P-AGTTGAGATCGGAAGAGCGGTTCAGCAGGAATGCCGAG	PE2-CAACT	ACACTCTTTCCCTACACGACGCTCTTCCGATCTCAACTT
PE1-CGCAT	P-ATGCGAGATCGGAAGAGCGGTTCAGCAGGAATGCCGAG	PE2-CGCAT	ACACTCTTTCCCTACACGACGCTCTTCCGATCTCGCATT
PE1-CTGGA	P-TCCAGAGATCGGAAGAGCGGTTCAGCAGGAATGCCGAG	PE2-CTGGA	ACACTCTTTCCCTACACGACGCTCTTCCGATCTCTGGAT
PE1-GCTTG	P-CAAGCAGATCGGAAGAGCGGTTCAGCAGGAATGCCGAG	PE2-GCTTG	ACACTCTTTCCCTACACGACGCTCTTCCGATCTGCTTGT
PE1-GTAAC	P-GTTACAGATCGGAAGAGCGGTTCAGCAGGAATGCCGAG	PE2-GTAAC	ACACTCTTTCCCTACACGACGCTCTTCCGATCTGTAACT
PE1-TCGGC	P-GCCGAAGATCGGAAGAGCGGTTCAGCAGGAATGCCGAG	PE2-TCGGC	ACACTCTTTCCCTACACGACGCTCTTCCGATCTTCGGCT
PE1-AACGA	P-TCGTTAGATCGGAAGAGCGGTTCAGCAGGAATGCCGAG	PE2-AACGA	ACACTCTTTCCCTACACGACGCTCTTCCGATCTAACGAT
PE1-ACGCT	P-AGCGTAGATCGGAAGAGCGGTTCAGCAGGAATGCCGAG	PE2-ACGCT	ACACTCTTTCCCTACACGACGCTCTTCCGATCTACGCTT
PE1-CTTAC	P-GTAAGAGATCGGAAGAGCGGTTCAGCAGGAATGCCGAG	PE2-CTTAC	ACACTCTTTCCCTACACGACGCTCTTCCGATCTCTTACT
PE1-GAATA	P-TATTCAGATCGGAAGAGCGGTTCAGCAGGAATGCCGAG	PE2-GAATA	ACACTCTTTCCCTACACGACGCTCTTCCGATCTGAATAT
PE1-GCCTT	P-AAGGCAGATCGGAAGAGCGGTTCAGCAGGAATGCCGAG	PE2-GCCTT	ACACTCTTTCCCTACACGACGCTCTTCCGATCTGCCTTT
PE1-GGTAG	P-CTACCAGATCGGAAGAGCGGTTCAGCAGGAATGCCGAG	PE2-GGTAG	ACACTCTTTCCCTACACGACGCTCTTCCGATCTGGTAGT
PE1-TGATC	P-GATCAAGATCGGAAGAGCGGTTCAGCAGGAATGCCGAG	PE2-TGATC	ACACTCTTTCCCTACACGACGCTCTTCCGATCTTGATCT
PE1-TGGCG	P-CGCCAAGATCGGAAGAGCGGTTCAGCAGGAATGCCGAG	PE2-TGGCG	ACACTCTTTCCCTACACGACGCTCTTCCGATCTTGGCGT
PE1-AGAAC	P-GTTCTAGATCGGAAGAGCGGTTCAGCAGGAATGCCGAG	PE2-AGAAC	ACACTCTTTCCCTACACGACGCTCTTCCGATCTAGAACT
PE1-AGATA	P-TATCTAGATCGGAAGAGCGGTTCAGCAGGAATGCCGAG	PE2-AGATA	ACACTCTTTCCCTACACGACGCTCTTCCGATCTAGATAT
PE1-CCTGA	P-TCAGGAGATCGGAAGAGCGGTTCAGCAGGAATGCCGAG	PE2-CCTGA	ACACTCTTTCCCTACACGACGCTCTTCCGATCTCCTGAT
PE1-CGGCT	P-AGCCGAGATCGGAAGAGCGGTTCAGCAGGAATGCCGAG	PE2-CGGCT	ACACTCTTTCCCTACACGACGCTCTTCCGATCTCGGCTT
PE1-GAGGC	P-GCCTCAGATCGGAAGAGCGGTTCAGCAGGAATGCCGAG	PE2-GAGGC	ACACTCTTTCCCTACACGACGCTCTTCCGATCTGAGGCT
PE1-GAGTT	P-AACTCAGATCGGAAGAGCGGTTCAGCAGGAATGCCGAG	PE2-GAGTT	ACACTCTTTCCCTACACGACGCTCTTCCGATCTGAGTTT
PE1-GTCAG	P-CTGACAGATCGGAAGAGCGGTTCAGCAGGAATGCCGAG	PE2-GTCAG	ACACTCTTTCCCTACACGACGCTCTTCCGATCTGTCAGT
PE1-TTCCG	P-CGGAAAGATCGGAAGAGCGGTTCAGCAGGAATGCCGAG	PE2-TTCCG	ACACTCTTTCCCTACACGACGCTCTTCCGATCTTTCCGT
PE1-AATAG	P-CTATTAGATCGGAAGAGCGGTTCAGCAGGAATGCCGAG	PE2-AATAG	ACACTCTTTCCCTACACGACGCTCTTCCGATCTAATAGT
PE1-ACCTA	P-TAGGTAGATCGGAAGAGCGGTTCAGCAGGAATGCCGAG	PE2-ACCTA	ACACTCTTTCCCTACACGACGCTCTTCCGATCTACCTAT
PE1-CGTCC	P-GGACGAGATCGGAAGAGCGGTTCAGCAGGAATGCCGAG	PE2-CGTCC	ACACTCTTTCCCTACACGACGCTCTTCCGATCTCGTCCT
PE1-CTGCC	P-GGCAGAGATCGGAAGAGCGGTTCAGCAGGAATGCCGAG	PE2-CTGCC	ACACTCTTTCCCTACACGACGCTCTTCCGATCTCTGCCT
PE1-GCTAT	P-ATAGCAGATCGGAAGAGCGGTTCAGCAGGAATGCCGAG	PE2-GCTAT	ACACTCTTTCCCTACACGACGCTCTTCCGATCTGCTATT
PE1-GGCGT	P-ACGCCAGATCGGAAGAGCGGTTCAGCAGGAATGCCGAG	PE2-GGCGT	ACACTCTTTCCCTACACGACGCTCTTCCGATCTGGCGTT
PE1-TAATG	P-CATTAAGATCGGAAGAGCGGTTCAGCAGGAATGCCGAG	PE2-TAATG	ACACTCTTTCCCTACACGACGCTCTTCCGATCTTAATGT
PE1-TTAGA	P-TCTAAAGATCGGAAGAGCGGTTCAGCAGGAATGCCGAG	PE2-TTAGA	ACACTCTTTCCCTACACGACGCTCTTCCGATCTTTAGAT
PE1-AAGCA	P-TGCTTAGATCGGAAGAGCGGTTCAGCAGGAATGCCGAG	PE2-AAGCA	ACACTCTTTCCCTACACGACGCTCTTCCGATCTAAGCAT
PE1-ACGGA	P-TCCGTAGATCGGAAGAGCGGTTCAGCAGGAATGCCGAG	PE2-ACGGA	ACACTCTTTCCCTACACGACGCTCTTCCGATCTACGGAT
PE1-CATAT	P-ATATGAGATCGGAAGAGCGGTTCAGCAGGAATGCCGAG	PE2-CATAT	ACACTCTTTCCCTACACGACGCTCTTCCGATCTCATATT
PE1-CCTAG	P-CTAGGAGATCGGAAGAGCGGTTCAGCAGGAATGCCGAG	PE2-CCTAG	ACACTCTTTCCCTACACGACGCTCTTCCGATCTCCTAGT
PE1-GGCTC	P-GAGCCAGATCGGAAGAGCGGTTCAGCAGGAATGCCGAG	PE2-GGCTC	ACACTCTTTCCCTACACGACGCTCTTCCGATCTGGCTCT
PE1-GTATT	P-AATACAGATCGGAAGAGCGGTTCAGCAGGAATGCCGAG	PE2-GTATT	ACACTCTTTCCCTACACGACGCTCTTCCGATCTGTATTT
PE1-TGAGG	P-CCTCAAGATCGGAAGAGCGGTTCAGCAGGAATGCCGAG	PE2-TGAGG	ACACTCTTTCCCTACACGACGCTCTTCCGATCTTGAGGT
PE1-TTACC	P-GGTAAAGATCGGAAGAGCGGTTCAGCAGGAATGCCGAG	PE2-TTACC	ACACTCTTTCCCTACACGACGCTCTTCCGATCTTTACCT
PE1-AAGGT	P-ACCTTAGATCGGAAGAGCGGTTCAGCAGGAATGCCGAG	PE2-AAGGT	ACACTCTTTCCCTACACGACGCTCTTCCGATCTAAGGTT
PE1-AGCTT	P-AAGCTAGATCGGAAGAGCGGTTCAGCAGGAATGCCGAG	PE2-AGCTT	ACACTCTTTCCCTACACGACGCTCTTCCGATCTAGCTTT
PE1-ATCTG	P-CAGATAGATCGGAAGAGCGGTTCAGCAGGAATGCCGAG	PE2-ATCTG	ACACTCTTTCCCTACACGACGCTCTTCCGATCTATCTGT
PE1-CATCG	P-CGATGAGATCGGAAGAGCGGTTCAGCAGGAATGCCGAG	PE2-CATCG	ACACTCTTTCCCTACACGACGCTCTTCCGATCTCATCGT
PE1-GCGAA	P-TTCGCAGATCGGAAGAGCGGTTCAGCAGGAATGCCGAG	PE2-GCGAA	ACACTCTTTCCCTACACGACGCTCTTCCGATCTGCGAAT
PE1-GGTCA	P-TGACCAGATCGGAAGAGCGGTTCAGCAGGAATGCCGAG	PE2-GGTCA	ACACTCTTTCCCTACACGACGCTCTTCCGATCTGGTCAT
PE1-GTCGC	P-GCGACAGATCGGAAGAGCGGTTCAGCAGGAATGCCGAG	PE2-GTCGC	ACACTCTTTCCCTACACGACGCTCTTCCGATCTGTCGCT
PE1-TCAAC	P-GTTGAAGATCGGAAGAGCGGTTCAGCAGGAATGCCGAG	PE2-TCAAC	ACACTCTTTCCCTACACGACGCTCTTCCGATCTTCAACT
PE1-AATCT	P-AGATTAGATCGGAAGAGCGGTTCAGCAGGAATGCCGAG	PE2-AATCT	ACACTCTTTCCCTACACGACGCTCTTCCGATCTAATCTT
PE1-AATGC	P-GCATTAGATCGGAAGAGCGGTTCAGCAGGAATGCCGAG	PE2-AATGC	ACACTCTTTCCCTACACGACGCTCTTCCGATCTAATGCT
PE1-AGCAG	P-CTGCTAGATCGGAAGAGCGGTTCAGCAGGAATGCCGAG	PE2-AGCAG	ACACTCTTTCCCTACACGACGCTCTTCCGATCTAGCAGT
PE1-CTGAG	P-CTCAGAGATCGGAAGAGCGGTTCAGCAGGAATGCCGAG	PE2-CTGAG	ACACTCTTTCCCTACACGACGCTCTTCCGATCTCTGAGT
PE1-GGAGC	P-GCTCCAGATCGGAAGAGCGGTTCAGCAGGAATGCCGAG	PE2-GGAGC	ACACTCTTTCCCTACACGACGCTCTTCCGATCTGGAGCT

PE1-GTCTA	P-TAGACAGATCGGAAGAGCGGTTCAGCAGGAATGCCGAG	PE2-GTCTA	ACACTCTTTCCCTACACGACGCTCTTCCGATCTGTCTAT
PE1-TAGCT	P-AGCTAAGATCGGAAGAGCGGTTCAGCAGGAATGCCGAG	PE2-TAGCT	ACACTCTTTCCCTACACGACGCTCTTCCGATCTTAGCTT
PE1-TCATA	P-TATGAAGATCGGAAGAGCGGTTCAGCAGGAATGCCGAG	PE2-TCATA	ACACTCTTTCCCTACACGACGCTCTTCCGATCTTCATAT
PE1-ACTCG	P-CGAGTAGATCGGAAGAGCGGTTCAGCAGGAATGCCGAG	PE2-ACTCG	ACACTCTTTCCCTACACGACGCTCTTCCGATCTACTCGT
PE1-AGTTG	P-CAACTAGATCGGAAGAGCGGTTCAGCAGGAATGCCGAG	PE2-AGTTG	ACACTCTTTCCCTACACGACGCTCTTCCGATCTAGTTGT
PE1-CGATT	P-AATCGAGATCGGAAGAGCGGTTCAGCAGGAATGCCGAG	PE2-CGATT	ACACTCTTTCCCTACACGACGCTCTTCCGATCTCGATTT
PE1-CGGAC	P-GTCCGAGATCGGAAGAGCGGTTCAGCAGGAATGCCGAG	PE2-CGGAC	ACACTCTTTCCCTACACGACGCTCTTCCGATCTCGGACT
PE1-GCAAG	P-CTTGCAGATCGGAAGAGCGGTTCAGCAGGAATGCCGAG	PE2-GCAAG	ACACTCTTTCCCTACACGACGCTCTTCCGATCTGCAAGT
PE1-GCCGA	P-TCGGCAGATCGGAAGAGCGGTTCAGCAGGAATGCCGAG	PE2-GCCGA	ACACTCTTTCCCTACACGACGCTCTTCCGATCTGCCGAT
PE1-GTTAA	P-TTAACAGATCGGAAGAGCGGTTCAGCAGGAATGCCGAG	PE2-GTTAA	ACACTCTTTCCCTACACGACGCTCTTCCGATCTGTTAAT
PE1-TACGC	P-GCGTAAGATCGGAAGAGCGGTTCAGCAGGAATGCCGAG	PE2-TACGC	ACACTCTTTCCCTACACGACGCTCTTCCGATCTTACGCT
PE1-ATCGT	P-ACGATAGATCGGAAGAGCGGTTCAGCAGGAATGCCGAG	PE2-ATCGT	ACACTCTTTCCCTACACGACGCTCTTCCGATCTATCGTT
PE1-ATTCC	P-GGAATAGATCGGAAGAGCGGTTCAGCAGGAATGCCGAG	PE2-ATTCC	ACACTCTTTCCCTACACGACGCTCTTCCGATCTATTCCT
PE1-CCAGC	P-GCTGGAGATCGGAAGAGCGGTTCAGCAGGAATGCCGAG	PE2-CCAGC	ACACTCTTTCCCTACACGACGCTCTTCCGATCTCCAGCT
PE1-CCGCA	P-TGCGGAGATCGGAAGAGCGGTTCAGCAGGAATGCCGAG	PE2-CCGCA	ACACTCTTTCCCTACACGACGCTCTTCCGATCTCCGCAT
PE1-GATAC	P-GTATCAGATCGGAAGAGCGGTTCAGCAGGAATGCCGAG	PE2-GATAC	ACACTCTTTCCCTACACGACGCTCTTCCGATCTGATACT
PE1-GGATG	P-CATCCAGATCGGAAGAGCGGTTCAGCAGGAATGCCGAG	PE2-GGATG	ACACTCTTTCCCTACACGACGCTCTTCCGATCTGGATGT
PE1-GTCCT	P-AGGACAGATCGGAAGAGCGGTTCAGCAGGAATGCCGAG	PE2-GTCCT	ACACTCTTTCCCTACACGACGCTCTTCCGATCTGTCCTT
PE1-TCGAT	P-ATCGAAGATCGGAAGAGCGGTTCAGCAGGAATGCCGAG	PE2-TCGAT	ACACTCTTTCCCTACACGACGCTCTTCCGATCTTCGATT
PE1-AGCGC	P-GCGCTAGATCGGAAGAGCGGTTCAGCAGGAATGCCGAG	PE2-AGCGC	ACACTCTTTCCCTACACGACGCTCTTCCGATCTAGCGCT
PE1-CCAAT	P-ATTGGAGATCGGAAGAGCGGTTCAGCAGGAATGCCGAG	PE2-CCAAT	ACACTCTTTCCCTACACGACGCTCTTCCGATCTCCAATT
PE1-CGCTG	P-CAGCGAGATCGGAAGAGCGGTTCAGCAGGAATGCCGAG	PE2-CGCTG	ACACTCTTTCCCTACACGACGCTCTTCCGATCTCGCTGT
PE1-CTAGT	P-ACTAGAGATCGGAAGAGCGGTTCAGCAGGAATGCCGAG	PE2-CTAGT	ACACTCTTTCCCTACACGACGCTCTTCCGATCTCTAGTT
PE1-TAGAG	P-CTCTAAGATCGGAAGAGCGGTTCAGCAGGAATGCCGAG	PE2-TAGAG	ACACTCTTTCCCTACACGACGCTCTTCCGATCTTAGAGT
PE1-TAGTC	P-GACTAAGATCGGAAGAGCGGTTCAGCAGGAATGCCGAG	PE2-TAGTC	ACACTCTTTCCCTACACGACGCTCTTCCGATCTTAGTCT
PE1-TATCA	P-TGATAAGATCGGAAGAGCGGTTCAGCAGGAATGCCGAG	PE2-TATCA	ACACTCTTTCCCTACACGACGCTCTTCCGATCTTATCAT
PE1-TTGCA	P-TGCAAAGATCGGAAGAGCGGTTCAGCAGGAATGCCGAG	PE2-TTGCA	ACACTCTTTCCCTACACGACGCTCTTCCGATCTTTGCAT
PE1-AACTC	P-GAGTTAGATCGGAAGAGCGGTTCAGCAGGAATGCCGAG	PE2-AACTC	ACACTCTTTCCCTACACGACGCTCTTCCGATCTAACTCT
PE1-CAAGG	P-CCTTGAGATCGGAAGAGCGGTTCAGCAGGAATGCCGAG	PE2-CAAGG	ACACTCTTTCCCTACACGACGCTCTTCCGATCTCAAGGT
PE1-CCGTT	P-AACGGAGATCGGAAGAGCGGTTCAGCAGGAATGCCGAG	PE2-CCGTT	ACACTCTTTCCCTACACGACGCTCTTCCGATCTCCGTTT
PE1-CTTCA	P-TGAAGAGATCGGAAGAGCGGTTCAGCAGGAATGCCGAG	PE2-CTTCA	ACACTCTTTCCCTACACGACGCTCTTCCGATCTCTTCAT
PE1-GTTCG	P-CGAACAGATCGGAAGAGCGGTTCAGCAGGAATGCCGAG	PE2-GTTCG	ACACTCTTTCCCTACACGACGCTCTTCCGATCTGTTCGT
PE1-TCAGT	P-ACTGAAGATCGGAAGAGCGGTTCAGCAGGAATGCCGAG	PE2-TCAGT	ACACTCTTTCCCTACACGACGCTCTTCCGATCTTCAGTT
PE1-TCCAA	P-TTGGAAGATCGGAAGAGCGGTTCAGCAGGAATGCCGAG	PE2-TCCAA	ACACTCTTTCCCTACACGACGCTCTTCCGATCTTCCAAT
PE1-TTGAC	P-GTCAAAGATCGGAAGAGCGGTTCAGCAGGAATGCCGAG	PE2-TTGAC	ACACTCTTTCCCTACACGACGCTCTTCCGATCTTTGACT

Oligonucleotides for adatpers with 3 nucleotide barcodes (8 in total)

Forward	Sequence	Reverse	Sequence2
PE1-ACC	P-GGTAGATCGGAAGAGCGGTTCAGCAGGAATGCCGAG	PE2-AAGAC	ACACTCTTTCCCTACACGACGCTCTTCCGATCTACCT
PE1-AGG	P-CCTAGATCGGAAGAGCGGTTCAGCAGGAATGCCGAG	PE2-ACCAT	ACACTCTTTCCCTACACGACGCTCTTCCGATCTAGGT
PE1-CAC	P-GTGAGATCGGAAGAGCGGTTCAGCAGGAATGCCGAG	PE2-AGACG	ACACTCTTTCCCTACACGACGCTCTTCCGATCTCACT
PE1-CTA	P-TAGAGATCGGAAGAGCGGTTCAGCAGGAATGCCGAG	PE2-CATTA	ACACTCTTTCCCTACACGACGCTCTTCCGATCTCTAT
PE1-GCT	P-AGCAGATCGGAAGAGCGGTTCAGCAGGAATGCCGAG	PE2-GCTGC	ACACTCTTTCCCTACACGACGCTCTTCCGATCTGCTT
PE1-GGA	P-TCCAGATCGGAAGAGCGGTTCAGCAGGAATGCCGAG	PE2-GTAGG	ACACTCTTTCCCTACACGACGCTCTTCCGATCTGGAT
PE1-TAT	P-ATAAGATCGGAAGAGCGGTTCAGCAGGAATGCCGAG	PE2-TGCCT	ACACTCTTTCCCTACACGACGCTCTTCCGATCTTATT
PE1-TTG	P-CAAAGATCGGAAGAGCGGTTCAGCAGGAATGCCGAG	PE2-TGGTA	ACACTCTTTCCCTACACGACGCTCTTCCGATCTTTGT

Supplementary Table 3

Barcode ID	Barcode	Average % reads mapped	Average reads
3-A-17	AACGA	75.19	6310417
3-B-18	ACGCT	76.02	4805973.5
3-C-19	CTTAC	71.70	4938993
3-D-20	GAATA	74.83	6806467.5
3-E-21	GCCTT	75.84	5785063
3-F-22	GGTAG	75.56	5182368.5
3-G-23	TGATC	75.18	6220377.5
3-H-24	TGGCG	75.68	5176310
4-A-25	AGAAC	76.15	6225416.5
4-B-26	AGATA	75.41	5182402.5
4-C-27	CCTGA	75.35	5312209.5
4-D-28	CGGCT	75.93	4505119
4-E-29	GAGGC	76.14	5671590.5
4-F-30	GAGTT	75.99	7014863.5
4-G-31	GTCAG	76.70	4746858.5
4-H-32	TTCCG	76.37	5427364
5-A-33	AATAG	75.63	6350079.5
5-B-34	ACCTA	73.00	5046391.5
5-C-35	CGTCC	75.90	4935055
5-D-36	CTGCC	75.92	4709005
5-E-37	GCTAT	75.91	4706736
5-F-38	GGCGT	78.02	6119736
5-G-39	TAATG	76.92	5866673.5
5-H-40	TTAGA	76.38	5712141.5
6-A-41	AAGCA	74.81	6544606.5
6-B-42	ACGGA	73.68	3785559.5
6-C-43	CATAT	75.93	5518970
6-D-44	CCTAG	75.96	6231612
6-E-45	GGCTC	77.11	5592509.5
6-F-46	GTATT	75.57	5392559
6-G-47	TGAGG	76.41	5239333.5
6-H-48	TTACC	76.06	4551023.5
7-A-49	AAGGT	76.06	7706802
7-B-50	AGCTT	75.83	5120243.5
7-C-51	ATCTG	75.09	6129435.5
7-D-52	CATCG	76.57	4949369.5
7-E-53	GCGAA	76.19	5702495.5
7-F-54	GGTCA	75.48	5647651
7-G-55	GTCGC	75.97	5522303.5
7-H-56	TCAAC	75.47	5059069
8-A-57	AATCT	75.82	4440916.5
8-B-58	AATGC	76.25	6670377
8-C-59	AGCAG	75.53	5594420.5
8-D-60	CTGAG	75.77	6939841.5

8-E-61	GGAGC	75.35	6506855
8-F-62	GTCTA	75.72	5082406
8-G-63	TAGCT	74.98	5776626.5
8-H-64	TCATA	72.09	3875830
9-A-65	ACTCG	75.71	3262272.5
9-B-66	AGTTG	76.08	5414195
9-C-67	CGATT	75.74	7801767
9-D-68	CGGAC	75.21	4462489.5
9-E-69	GCAAG	75.74	6063922.5
9-F-70	GCCGA	75.42	5668124
9-G-71	GTTAA	75.80	6381386.5
9-H-72	TACGC	75.98	6238605
10-A-73	ATCGT	75.87	6082062.5
10-B-74	ATTCC	77.59	6029126.5
10-C-75	CCAGC	77.02	4496062
10-D-76	CCGCA	75.93	4787150.5
10-E-77	GATAC	76.59	4937230
10-F-78	GGATG	75.07	3968972.5
10-G-79	GTCCT	76.09	4220117.5
10-H-80	TCGAT	75.08	4522526
11-A-81	AGCGC	76.39	3610090.5
11-B-82	CCAAT	76.70	4878664
11-C-83	CGCTG	75.85	5493921
11-D-84	CTAGT	75.52	4480073.5
11-E-85	TAGAG	77.53	5472796.5
11-F-86	TAGTC	76.41	5322538.5
11-G-87	TATCA	75.91	5102129.5
11-H-88	TTGCA	75.59	5790716
12-A-89	AACTC	77.36	5441668
12-B-90	CAAGG	75.66	4894013.5
12-C-91	CCGTT	76.42	5256868
12-D-92	CTTCA	76.07	5778642.5
12-E-93	GTTCG	76.63	4366941.5
12-F-94	TCAGT	76.16	5403931.5
12-G-95	TCCAA	75.26	5574095.5
12-H-96	TTGAC	76.69	6130274

Supplementary Table 4

Library	Reads post filter	% Reads post-filter
M11	6253353	98.52
M12	4199127	99.23
M13	3008295	99.07
M15	5936521	97.49
P11	4810008	98.69
P12	4499977	98.80
P13	3517571	99.02
P15	4186029	97.66
M41	3635645	98.61
M42	3099471	98.75
M43	4355405	97.84
M45	3452309	99.24
P41	5571477	97.27
P42	3564549	98.69
P43	4004353	98.61
P45	2276871	99.13

Supplementary Table 5

Category	IL_SLY	IL_SPE	HTR_SLY	HTR_SPE
(a) SLY down only in HTR	171.26	170.17	113.68	154.74
(b) SLY up only in HTR	197.24	184.06	306.20	183.32
(c) SLY down in IL and up in HTR	374.81	508.01	513.93	332.70
(d) SLY down in both IL and HTR	107.60	175.27	99.90	171.25
(e) SLY up in both IL and HTR	242.18	129.92	284.72	118.58
(f) SLY down only in IL	147.55	203.24	209.79	192.08
(g) SLY up only in IL	186.83	122.35	124.09	110.51
(h) SLY up in IL and down in HTR	382.41	264.56	155.48	204.52

Supplementary Table 6

Comparison	Group1a	Group1b	Group1_DE	Group2a	Group2b	Group2_DE	Common_DE	Unique_DE	All_DE	%_Common	%_Unique
IL vs HTR	M11,M12,M13,M15	P11,P12,P13,P15	6620	M41,M42,M43,M45	P41,P42,P43,P45	10326	5032	6882	11914	42.24	57.76
IL vs HTR	M11,M12	P11,P12	4115	M41,M42	P41,P42	6988	2977	5149	8126	36.64	63.36
IL vs HTR	M12,M13	P12,P13	4449	M42,M43	P42,P43	6808	2949	5359	8308	35.50	64.50
IL vs HTR	M13,M15	P13,P15	3820	M43,M45	P43,P45	7377	2770	5657	8427	32.87	67.13
IL vs IL	M11,M12	P11,P12	4115	M13,M15	P13,P15	3820	2828	2279	5107	55.37	44.63
IL vs IL	M11,M12	P11,P13	4076	M13,M15	P12,P15	3666	2761	2220	4981	55.43	44.57
IL vs IL	M11,M12	P11,P15	3981	M13,M15	P12,P13	4131	2784	2544	5328	52.25	47.75
IL vs IL	M11,M13	P11,P12	3982	M12,M15	P13,P15	3610	2685	2222	4907	54.72	45.28
IL vs IL	M11,M13	P11,P13	3899	M12,M15	P12,P15	3545	2654	2136	4790	55.41	44.59
IL vs IL	M11,M13	P11,P15	3790	M12,M15	P12,P13	3900	2662	2366	5028	52.94	47.06
IL vs IL	M11,M15	P11,P12	3443	M12,M13	P13,P15	4153	2637	2322	4959	53.18	46.82
IL vs IL	M11,M15	P11,P13	3385	M12,M13	P12,P15	3991	2576	2224	4800	53.67	46.33
IL vs IL	M11,M15	P11,P15	3329	M12,M13	P12,P13	4449	2581	2616	5197	49.66	50.34
HTR vs HTR	M41,M42	P41,P42	6988	M43,M45	P43,P45	7377	5599	3167	8766	63.87	36.13
HTR vs HTR	M41,M42	P41,P43	7016	M43,M45	P42,P45	7416	5643	3146	8789	64.21	35.79
HTR vs HTR	M41,M42	P41,P45	7103	M43,M45	P42,P43	7349	5593	3266	8859	63.13	36.87
HTR vs HTR	M41,M43	P41,P42	6368	M42,M45	P43,P45	7345	5304	3105	8409	63.08	36.92
HTR vs HTR	M41,M43	P41,P43	6457	M42,M45	P42,P45	7432	5435	3019	8454	64.29	35.71
HTR vs HTR	M41,M43	P41,P45	6437	M42,M45	P42,P43	7302	5360	3019	8379	63.97	36.03
HTR vs HTR	M41,M45	P41,P42	6707	M42,M43	P43,P45	6799	5364	2778	8142	65.88	34.12
HTR vs HTR	M41,M45	P41,P43	6826	M42,M43	P42,P45	6850	5388	2900	8288	65.01	34.99
HTR vs HTR	M41,M45	P41,P45	6807	M42,M43	P42,P43	6808	5365	2885	8250	65.03	34.97
